# Implementation of a best-practice model of care for cognitive impairment and dementia for first nations peoples attending primary care in Australia: a stepped-wedge cluster-randomised trial

**DOI:** 10.1016/j.lanwpc.2025.101529

**Published:** 2025-04-03

**Authors:** Jo-anne Hughson, Zoë Hyde, Kate Bradley, Roslyn Malay, Harold Douglas, Sadia Rind, Kylie Sullivan, Lauren Poulos, Bridget Allen, Bonnie Martin-Giles, Rachel Quigley, Sarah Russell, Diane Cadet-James, Valda Wallace, Wendy Allan, Dawn Bessarab, Kate Smith, Kylie Radford, Edward Strivens, Leon Flicker, David Atkinson, Sandra Thompson, Juliette Ciaccia, Louise Lavrencic, Belinda Ducker, Tina Humphry, Mark Wenitong, Mary Belfrage, Irene Blackberry, Kate Fulford, Sharon Wall, Robyn Smith, Dina LoGiudice

**Affiliations:** a Department of Medicine – Royal Melbourne Hospital, The University of Melbourne, Royal Park Campus, Administration Building 21, 34-54 Poplar Road, Melbourne, Victoria 3052, Australia; b Western Australian Centre for Heath and Ageing, Medical School, The University of Western Australia, 35 Stirling Highway, Perth, WA 6009, Australia; c Centre for Aboriginal Medical and Dental Health, Medical School, The University of Western Australia, 35 Stirling Highway, Perth, WA 6009, Australia; d Derbarl Yerrigan Health Service, 22 Chesterfield Rd, Mirrabooka, WA 6061, Australia; e Neuroscience Research Australia, 139 Barker Street, Sydney, NSW 2031, Australia; f John Richards Centre, La Trobe Rural Health School, La Trobe University, PO Box 821, Wodonga, VIC 3689, Australia; g Healthy Ageing Research Team, James Cook University, Nguma-bada campus, Smithfield, QLD 4878, Australia; h Cairns and Hinterland Hospital and Health Service, Queensland, Australia; i School of Psychology, The University of New South Wales, Sydney, Australia; j The Rural Clinical School of Western Australia, The University of Western Australia, PO Box 1377, Broome, WA 6725, Australia; k The Western Australian Centre for Rural Health, The University of Western Australia, 167 Fitzgerald St, Geraldton, WA 6530, Australia; l School of Population Health, University of New South Wales, Sydney, Australia; m Ageing Futures Institute, University of New South Wales, Sydney, Australia; n Care Economy Research Institute, La Trobe University, Wodonga, Australia; o Department of Aged Care, Royal Melbourne Hospital, Melbourne, Australia

**Keywords:** Dementia, Cognitive impairment not dementia, Aboriginal and Torres Strait Islander, Aboriginal community controlled health services, Models of care

## Abstract

**Background:**

Dementia and cognitive impairment not dementia (CIND) are under-detected amongst First Nations peoples attending primary care. This trial implemented a culturally adapted best-practice model of care to increase detection and optimise management of CIND/dementia.

**Methods:**

This closed cohort open-label, stepped-wedge, cluster-randomised trial recruited 12 Aboriginal community-controlled primary health care services (ACCHSs) across urban, regional and remote settings in Australia. ACCHSs were eligible to participate if they conducted annual health checks, engaged in continuous quality improvement processes and had ≥55 clients aged ≥50 years. After a baseline control period, four ACCHSs were scheduled to enter the intervention phase every six months. During the intervention phase, ACCHSs were supported to embed best-practice dementia care through staff education and practice change initiatives. Co-primary outcomes were: (i) documented detection of CIND/dementia and, (ii) evidence of uptake of the diagnostic pathway measured as presence of ≥2 of: use of cognitive assessment tools, relevant pathology investigations, neuroimaging, and/or referral of clients with cognitive concerns to specialist services. Data were analysed with mixed effects complementary log–log regression. This study was registered with the Australia and New Zealand Clinical Trials Registry, ACTRN12618001485224.

**Findings:**

Between September 2018 and January 2019, 12 ACCHSs were recruited, comprising a sample of 1655 ACCHS clients aged ≥50 years (mean 60.3 ± 8.2 years), of whom 935 (56.5%) were female. One ACCHS withdrew during the study. After adjustment for time, the intervention did not show evidence of an effect for the first co-primary outcome (detection of CIND/dementia): HR = 1.53 (95% CI 0.64, 3.65). However, the intervention improved the second co-primary outcome (uptake of diagnostic pathway): HR = 2.34 (95% CI 1.05, 5.25). Intention-to-treat analyses yielded similar results.

**Interpretation:**

The co-developed best-practice model of care for cognitive impairment and dementia for Aboriginal and Torres Strait Islander people attending primary care improved the diagnostic CIND/dementia management process.

**Funding:**

10.13039/501100000925National Health and Medical Research Council (Australia) and Dementia Training Australia.

**Research in context**

**Evidence before this study**

This team has demonstrated high rates of cognitive impairment and dementia in First Nations communities in Australia—up to five times those of the general population—across urban, rural and remote settings. While dementia types are comparable to the wider Australian population, age of onset is younger, late or under-detection is common and the risk factor profile is distinct. These findings highlighted the need for models of care designed with First Nations communities, including early detection strategies.

In February 2017, we systematically reviewed literature on primary care interventions to improve detection of cognitive impairment and dementia, as well as primary care interventions with a focus on First Nations peoples in relation to other health issues (e.g., diabetes management). We searched PubMed for English language publications using variations and different combinations of the terms “Aboriginal”, “dementia”, “primary care” and “model of care”. A systematic review on dementia detection and management in primary care found GP education improved detection rates. Other features of successful interventions were also identified. Efficacious primary care interventions in First Nations communities emphasised cultural safety, utilising First Nations researchers and health workers, local health service project ownership, and Continuous Quality Improvement (CQI) frameworks.

**Added value of this study**

An updated literature search in December 2024 did not identify any other interventions to improve detection and/or management of cognitive impairment and dementia in First Nations populations attending primary care in Australia, or internationally. As such, this study is a unique and foundational intervention, co-developing and trialling a best-practice model of care for cognitive impairment and dementia for First Nations communities attending primary care in Australia.

The program developed clinical and educational resources that are now publicly available, including the first dementia best-practice guide for this population. No impact on documentation of cognitive impairment and dementia was observed, but the intervention more than doubled the use of best-practice dementia assessment strategies.

**Implications of all the available evidence**

Co-development and implementation of a best-practice model of care for older First Nations peoples in Australia with cognitive impairment and dementia does improve uptake of the best-practice care pathway for detection. Further research is required to determine whether the observed improvement in cognitive assessment diagnostic practices can translate into increased detection of cognitive impairment and dementia. A follow-up audit will track potential effects post-intervention and assess sustainment. Study findings and feedback from key stakeholders will inform future interventions to improve the detection and management of cognitive impairment and dementia in First Nations communities.

## Introduction

Older Aboriginal and Torres Strait Islander (hereafter respectfully referred to as First Nations) people of Australia play a central role in the health and wellbeing of their families and communities, providing leadership and support, being the keepers of lore and cultural knowledges, and upholding connection to Country.^1^ Examples of older First Nations peoples ageing well abound, with more people living to older ages.^2^ Yet, on average, and similar to other Indigenous populations impacted by colonisation worldwide, in Australia First Nations peoples experience lower life expectancy (8.8 years lower for men and 8.1 years lower for women) and a burden of disease more than twice that of non-First Nations people.^3^ Dementia prevalence is three to five times higher in First Nations populations than the general Australian population.^4^, ^5^, ^6^

In Australia, types of dementia in First Nations peoples are comparable with the wider population, but the age of disease onset is considerably younger,^6^^,^^7^ and the risk factor profile is distinct. In some studies, First Nations men appear at greater risk, and head injury has been noted to play a greater role,^4^^,^^5^^,^^8^ with differing risk factor profiles seen in different communities.^8^ For First Nations peoples there is a higher cumulative life-course exposure to modifiable risks for dementia.^9^ Dementia risk increases with age. With the number of older First Nations peoples in Australia projected to increase by between 200% (60–64-year-old age group) and 800% (85-plus age group) by mid-century,^2^ initiatives addressing preventative strategies for and optimal management of dementia are crucial.

Prior research in the Kimberley region found that only 38% of First Nations peoples receiving a clinical diagnosis of dementia during the study had previously been diagnosed.^10^ Lack of training, confidence and dementia knowledge as well as competing healthcare priorities were health service barriers to care reported in two recent studies.^11^^,^^12^ Limited community awareness, denial, stigma, competing community priorities, distrust of mainstream services and the biomedical model, lack of culturally appropriate services, language barriers, and fear of being moved off Country were cited community barriers.^11^^,^^12^ Improving detection and management of dementia are priorities for First Nations dementia research identified by stakeholders, including 253 community members.^13^ Health professional education (in particular of GPs) has been found to improve dementia and cognitive impairment detection.^14^ Successful interventions are also associated with: decision support systems; availability of guidelines; and one-to-one and group education.^15^^,^^16^ A review on effectiveness of implementation in First Nations Australian healthcare found an emphasis on cultural safety (described in Belfrage et al.^17^^(p6)^), utilising First Nations researchers and health workers, and local health service project ownership to be key facilitators of implementation.^18^ Continuous Quality Improvement (CQI) programs are increasingly used by First Nations primary health care services and show promising improvements in care received by health service clients.^19^ CQI is a structured organisational process to improve health services that involves personnel in planning changes and then iteratively testing and refining the changes.^19^

Aboriginal Community Controlled Health Services (ACCHSs) are founded on the principles of self-determination and providing culturally safe, community-based and holistic primary healthcare.^20^ Approximately half the First Nations population nationally attends ACCHSs,^21^ valuing them as accessible, welcoming social spaces.^20^ As such, ACCHSs have a critical role in facilitating timely detection and management of cognitive impairment and dementia.

There are many reasons therefore to embed culturally appropriate and tailored best-practice models of dementia care in primary care settings. The Let’s CHAT (Community Health Approaches To) Dementia in Aboriginal and Torres Strait Islander Communities study implemented a culturally responsive best-practice model of dementia care with the primary aims of improving detection and management of dementia and cognitive impairment not dementia (CIND) among older First Nations peoples attending ACCHSs.^10^ The model was co-designed with ACCHS partners to ensure culturally safe and respectful research practice and to enable tailoring to individual ACCHSs and incorporation of Indigenous worldviews. A stepped-wedge cluster randomised trial (SWCRT) was chosen as an efficient and pragmatic design for evaluating service delivery interventions, and for the ethical and equitable feature of all participants experiencing the intervention.^22^ The primary hypotheses of this study were that implementation of the Let’s CHAT Dementia model of care and health system changes would result in an increase in documentation of dementia and CIND, and increased cognitive assessment and management for CIND/dementia in the clinic populations.

## Methods

### Study design and clusters

**Fig. 1:**
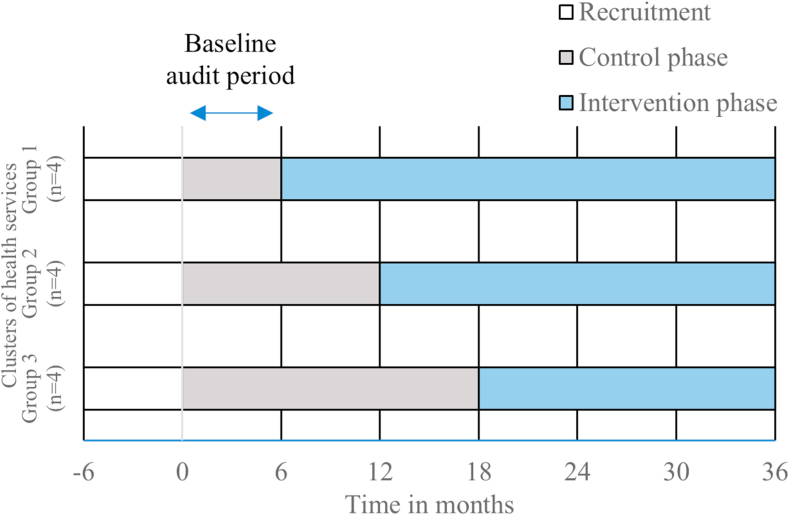
Schematic of the Let’s CHAT Dementia stepped-wedge cluster-randomised trial.

This study monitored the implementation of a best-practice model of care for CIND/dementia for First Nations people attending 12 ACCHSs in Australia using a SWCRT design with repeat measurements on the same cohort of individual clients.^22^ The model of care was founded on a clinical best-practice guide (BPG) developed for the project through a process of expert consensus to combine clinical guidelines with key cultural and clinical considerations.^23^ The Let’s CHAT implementation at each site comprised: (i) educational workshops facilitated by project team members—two for general practitioners (GPs) and six for other ACCHSs staff (including Aboriginal health workers/practitioners (AHW/Ps), nurses, allied health and aged care practitioners, and other personnel); (ii) access to the BPG and other resources; and (iii) continuous quality improvement (CQI) activities to support and evaluate practice change that were agreed within each ACCHS and facilitated by an appointed support person/liaison for the project within each ACCHS, referred to as the study’s ‘ageing well champion.’ Clusters entered the implementation phase six-monthly following a baseline control period (Fig. 1). Each co-researching ACCHS was expected to participate in the implementation phase of the study for at least 18 months, with the trial end scheduled when the third group reached 18 months of implementation.

We recruited ACCHSs across urban, regional and remote settings in four Australian states: New South Wales, Queensland, Victoria, and Western Australia. ACCHSs were eligible to participate if they: engaged in annual health checks (a formal government funded initiative); engaged in CQI processes; and had at least 55 clients aged ≥ 50 years. For over 20 years the investigator team has been working with First Nations communities on dementia care research and has strong links with communities. For recruitment, we approached ACCHSs with whom we had previously collaborated, state-level ACCHS peak bodies and other potential ACCHSs. Key research staff engaged in discussions with individual ACCHSs with follow-up expressions of interest documents describing the project and what involvement would entail. Resourcing commitments and remuneration (when requested) were agreed. Co-researching ACCHSs provided letters of support to formalise participation and formal ethics applications were made to ensure culturally appropriate governance, research process, data and intellectual property management.

This study was approved by the Aboriginal Health and Medical Research Committee of NSW (ref. 1362/18 and 1855/21), the University of Melbourne Human Research Ethics Committee (HREC) (ref. 1851943 and 12140), the Western Australian Aboriginal Health Ethics Committee (ref. 858), the Kimberley Aboriginal Health Planning Forum (ref. 2018-006) and James Cook University HREC (ref. H7371). Ethics approvals obtained for this study allowed for a waiver of individual informed consent for the audit data collection on the grounds that: (i) the information being sought was routinely collected in the primary care context as part of regular clinical audits, and; (ii) it was impracticable to seek individual consent from each client given the sample size, geographical remoteness of some ACCHS clients, and need to maintain the integrity of the sample across the data collection period. Partner ACCHSs authorised the use of routine clinical data for this research project only, under strict conditions of confidentiality, assurance of deidentification of individuals, and secure data storage by the research team. Given that the study had minimal risk of harm to ACCHS clients, there was no specific data monitoring committee. A culturally appropriate research governance model was adopted, with all aspects of the project overseen by an Indigenous Reference Group (IRG). The IRG comprised representatives from each state involved in the project (Vic, NSW, Qld, WA) who had experience as advocates for First Nations people and dementia—as a carer, health care worker and/or community member. ACCHSs and IRG members worked with the research team (which included First Nations and non-First Nations personnel) to co-design, plan and develop the implementation program and resources, data collection, analysis and dissemination work. All non-First Nations research personnel were required to undertake cultural awareness and/or safety training prior to starting their roles. This study was registered with the Australia and New Zealand Clinical Trials Registry (ACTRN12618001485224).

### Randomisation and blinding

A statistician external to the research project randomly assigned one cluster from each state to one of three cluster groups using simple randomisation. Investigators and research personnel were not blinded to the intervention. A few weeks prior to intervention commencement, Group 1 ACCHSs were advised they would be entering the intervention. Allocation concealment was ensured for Group 2 and Group 3 ACCHSs until immediately prior to Group 2 ACCHSs entering the intervention phase, at which point all ACCHSs were aware of their allocation sequence.

### Procedures

The Let’s CHAT Dementia intervention program has been described in detail previously.^10^ Due to the COVID-19 pandemic, it was not possible for the services to enter in groups of four after the first stage as planned. Individual ACCHSs entered at the first available opportunity with respect to their originally designated entry point. Research personnel (JH, HD, KB, BMG, DLG, BA, LP, BD, KR, WA, KSu, LL, SW, RQ, SRu, DCJ, VW, ES, RM, SRi, TH, KF, LF), including one or more Aboriginal Project Officers in each state team, carried out data collection and facilitated the implementation at each co-researching ACCHS. Once an ACCHS entered the intervention phase, educational workshops were scheduled at times and intervals convenient to the ACCHS. Workshops provided an opportunity to communicate information and provide resources around best-practice care for cognitive impairment and dementia. ACCHS staff were engaged in discussions, shared their ideas, priorities and strategies for initiating practice changes. Local research teams maintained regular contact with services throughout the intervention phase and provided support to local initiatives. ACCHSs were tasked with selecting ageing well champions to assist with implementing the model of care. Co-designed practice initiatives developed and implemented as part of the CQI process are described in the Panel 1.

Panel 1 **Best practice cognitive impairment and dementia care program and practice change initiatives for older First Nations peoples attending primary care**

All-staff Workshop Topics.

1. Detection of Cognitive Impairment and Dementia
2. Caring for People Living with Cognitive Impairment and Dementia
3. Health Promotion and Prevention
4. The Lived Experience, Building Empathy and Understanding
5. Health and Wellbeing of Carers of People with Cognitive Impairment and Dementia
6. Planning, Decision-making and End-of-life Care

GP Workshop Topics.

1. Detection, Diagnosis and Co-morbidity
2. Management of Cognitive Impairment and Dementia

Practice Change Initiatives.

• Updates to the Older Person’s Annual Health Check to include memory and thinking (completed with 6/11 ACCHSs)
• Setting up in-house specialist geriatrician/memory clinic (4/9 ACCHSs + already in place in 2 ACCHSs)
• Co-development of customised cognitive impairment and dementia protocol resource with local supports and services (7/11 ACCHSs)
• Provision of best-practice guide (11/11 ACCHSs)
• Provision of GP Management Plan (GPMP) recommendations (11/11 ACCHSs)
• Awareness raising with local First Nations community, including: Elders workshops, brain health video campaigns, creation of posters, social media tiles (8/11 ACCHSs)
• Kimberley Indigenous Cognitive Assessment Tool training workshops conducted with health service staff (6/11 ACCHSs)
• Ageing well champion roles established in the participating ACCHSs (recruited at 10/11 ACCHSs and retained for ≥75% of the study at 5/11 ACCHSs)

**Fig. 2:**
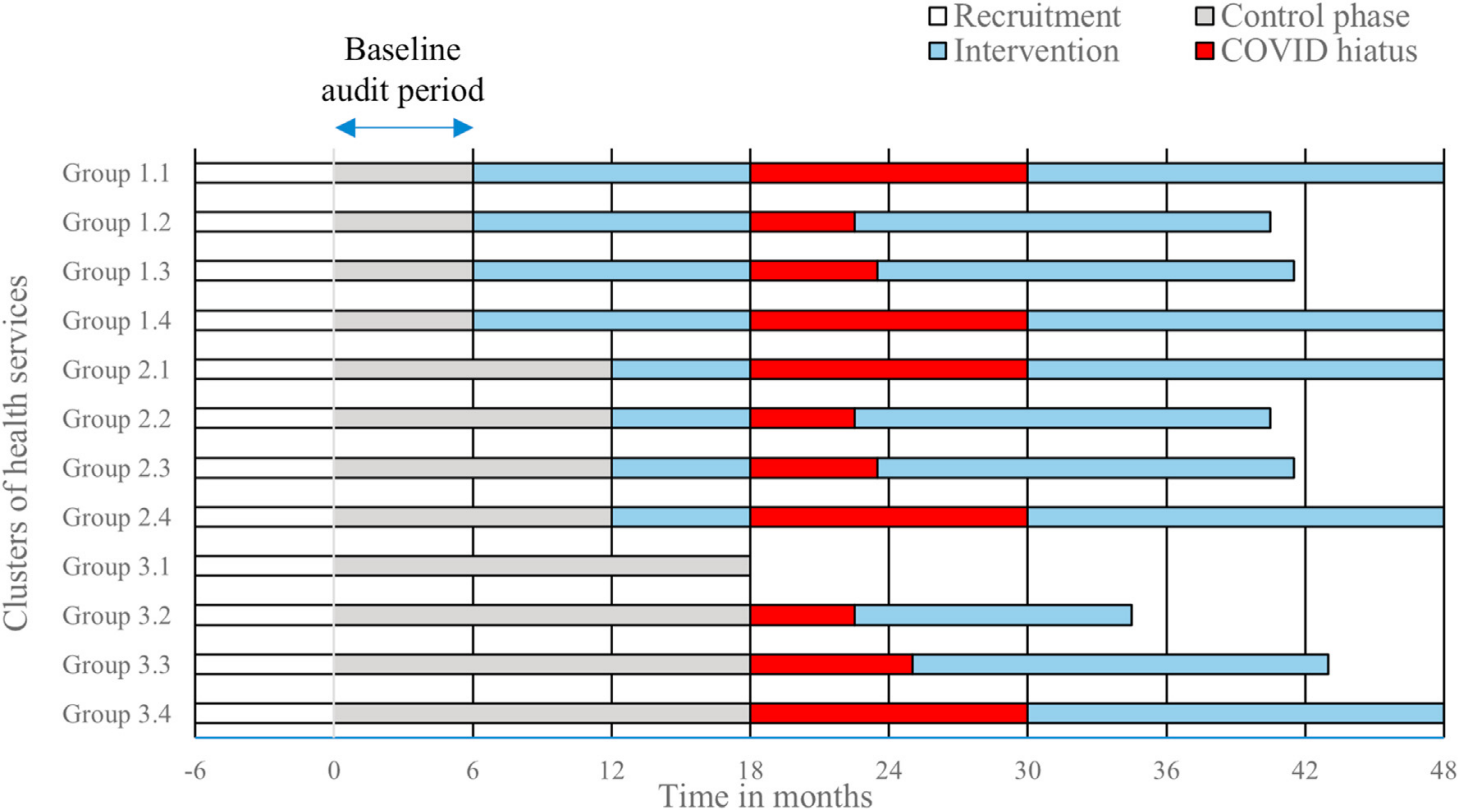
Schematic of the modified Let’s CHAT Dementia stepped-wedge cluster-randomised trial, post COVID-19.

Baseline audits of client medical records were conducted and thereafter planned six-monthly for a total period of three years. The audit tool was developed to monitor documented aspects of cognition-related client care (details reported elsewhere) and also to enable collection of demographic data as entered into the ACCHSs’ medical record systems.^10^ From mid-March 2020 the audit schedule was interrupted and the intervention halted at all sites for various periods (up to one year), depending on ACCHS location, due to the COVID-19 pandemic. A ‘COVID hiatus’ audit was retrospectively conducted for each site as soon as possible when research access resumed. Six-monthly auditing also resumed, with an altered study timeline (see Fig. 2). Natural disasters (bushfires and floods), sorry business, and travel restrictions relating to Aboriginal cultural law also impacted access to study sites. As a result of the disruptions, program adaptations were sometimes necessary, including transitioning to online delivery of education sessions in some services. In two remotely located ACCHSs, the implementation program was not able to be fully completed. Significant internal disruptions, directly related to understaffing and likely indirectly connected to the pandemic, meant that education and practice change work was not feasible later in the intervention for some ACCHSs. The full program was rolled out in the other nine ACCHSs, but fidelity to the timeline was not observed, with high implementation activity generally occurring towards the end of the project. This meant many ACCHS staff received more input on best-practice dementia care towards the end of the study.

To maximise data completeness and accuracy, a three-step data checking and cleaning procedure for each audit was developed in collaboration with the study biostatistician: (i) manual checking of dataset in Microsoft Excel to identify missing variables and errors; (ii) spot checks with full re-audits of randomly selected records for five clients at each ACCHS (two medical records of clients with identified concerns around cognition/confirmed cognitive impairment and three without); and (iii) automated checking for outliers and unexpected missing data. Data checking and cleaning was carried out by research personnel who had not conducted the audit being checked. All data inconsistencies and errors found were amended prior to statistical analysis.

As part of the CQI process, feedback was provided to ACCHSs in the form of audit summaries after baseline audits had been completed, at the conclusion of audit 3 or at the end of 2021, and after the final audit was conducted. Summaries documented individual ACCHS progress on outcome measures for the study and described project activities and practice change initiatives. Regular project newsletters detailed study progress and featured events/activities at individual ACCHSs.

### Outcomes

The co-primary outcomes were: (i) detection of CIND/dementia; and, (ii) evidence of uptake of the diagnostic pathway for CIND/dementia. Presence of CIND/dementia included a diagnosis of a neurocognitive disorder, such as Alzheimer’s disease or another type of dementia, mild cognitive impairment (MCI), or confirmed cognitive impairment but no diagnosis. During auditing, a condition qualified as a diagnosis if it was listed in the ‘diagnoses’ section of the client’s health record, or explicitly defined as such in correspondence found in the client’s record from a specialist. The second co-primary outcome included concerns being raised regarding cognition (by the client themselves, a member of the health care team or other person), and documentation in the client’s medical record of at least two of the following:

• cognitive assessment;
• relevant pathology investigations;
• relevant neuroimaging (CT or MRI); or
• referral to a memory clinic/geriatrician.

### Sample

The sampling frame comprised First Nations ACCHS clients aged ≥50 years. Clients were excluded if they were: extremely unwell and/or with likelihood of death within six months; not resident in the area for the previous 12 months; in residential aged care; and/or not active clients of the health service (defined as <3 clinic visits in the two years preceding study commencement). We aimed to audit 150 people from each health service (at ACCHSs with ≤150 patients meeting eligibility criteria, all records were audited), and subsequently audited the medical records of 1655 people. Through simulation of mixed effects complementary log–log models^24^ with an average cluster size of 138, coefficient of variation of 0.16, and total of 60 events, we determined that we had 16% power given a hazard ratio of 2.0 and alpha of 5%. With 120 events, we would have had 54% power given a hazard ratio of 2.0, and 80% power given a hazard ratio of 2.5.

### Statistical analysis

We used Stata version 17.0 (StataCorp, College Station, Texas) to analyse the data. We tested for baseline differences in client characteristics between the participating ACCHSs with Welch’s ANOVA in the case of continuous variables, and Pearson’s chi-squared test for categorical variables. Outcomes were explored with mixed effects complementary log–log regression,^24^ with an exchangeable correlation structure for the random effects. We planned to model variation between clusters with a random intercept effect, with time nested within clusters as a random coefficient.^10^ However, the planned models did not converge, presumably because fewer events occurred during the trial than expected. Accordingly, our models included only random intercepts for both participants and clusters. Intervention and time (number of months since the start of the trial) were included in the models as fixed effects. As these were survival models, individuals who had already experienced an outcome at baseline were excluded from analyses. Because we had the exact date of diagnosis of cognitive impairment for most events, we also analysed the first co-primary outcome with a Cox proportional hazards model with gamma-distributed shared frailty. A time-varying dichotomous variable recorded intervention status, with time-at-risk (measured in weeks) defined in each of the control and intervention periods for each person.^25^ Entry time was set to the start of the control period or intervention period (if applicable). Exit time was set to either the date of diagnosis, date of loss to follow-up, or the end of the control or intervention period, as appropriate. Where the diagnosis date was missing, it was set to the middle of the audit period in which the diagnosis was known to have occurred. We tested the Schoenfeld and martingale residuals to ensure the proportional hazards and linearity assumptions were met. We did not consider competing risks. We initially performed an intention-to-treat analysis, and subsequently a per-protocol analysis in which one health service that did not complete the trial was excluded, and one health service experienced the intervention later than planned. All statistical tests were two-tailed and we considered *p* values <0.05 statistically significant.

### Role of the funding source

The study funder had no role in study design, data collection, analysis, interpretation, or writing of the report.

## Results

Twelve ACCHSs across four states of Australia (three each in New South Wales, Queensland, Victoria and Western Australia) and across urban, regional and remote settings were recruited between September 2017 and June 2018. The study period ran from October 2018 to January 2023 (modified due to COVID-19 disruptions from the original end date of January 2022). Eleven ACCHSs completed the intervention (Supplementary Fig. S1). One service withdrew after completing three control audit periods and was excluded from the per-protocol analysis. Data from a total of 1655 clients were available for analysis at the start of the study. Sixty-seven had been diagnosed with cognitive impairment prior to the start of the study and were excluded from analyses. Of the remainder, 60 developed cognitive impairment during the trial, 913 were followed until the end of the trial, 283 until their last documented visit to their ACCHS, 97 until death, 117 until they left their ACCHS or were otherwise lost to follow-up, and 118 until their ACCHS withdrew from the trial (Table 1 and Supplementary Fig. S2).

**Table 1:** Number of participants remaining in the trial at each audit period.

Note: Shaded areas denote intervention period. Periods marked with an asterisk indicate disruption due to the COVID-19 pandemic.

NA = not applicable.

The mean age of clients at each ACCHS varied from 58.7 to 62.1 years (*p* = 0.0003), with an overall mean and standard deviation of 60.3 ± 8.2 years. The proportion of male and female clients was similar across each ACCHS (*p* = 0.40). Overall, 56.5% of clients (n = 935) were female. Sixty-seven clients (4.0%) had documented CIND/dementia at the start of the trial, and 55 clients (3.3%) met criteria for appropriate CIND/dementia management. By the end of the trial, the number of clients with documented cognitive impairment and appropriate CIND or dementia management had risen to 127 (7.7%) and 153 (9.2%), respectively (Supplementary Fig. S3). Changes in prevalence of components of the second co-primary outcome between baseline and the end of the trial were: concerns raised (9.6% to 20.5%); cognitive assessment tools used (12.6% to 23.8%); laboratory investigation (4.1% to 7.6%); imaging (4.6% to 12.8%); and referral to specialist (2.8% to 8.8%).

### Intention-to-treat analysis

Over a median (interquartile range [IQR]) 42 (30) months of follow-up, 60 people experienced the first co-primary outcome during the trial (documentation of CIND/dementia diagnosis), and 98 people experienced the second co-primary outcome (documented evidence of appropriate CIND/dementia diagnostic pathways). After adjustment for time, there was no evidence that the intervention led to an increase in the first co-primary outcome (HR = 1.40; 95% CI 0.56, 3.51; *p* = 0.47). Results were similar in a continuous-time survival analysis approach (Table 2). However, the intervention led to a significant increase in the second co-primary outcome (HR = 2.57; 95% CI 1.11, 5.96; *p* = 0.027).

**Table 2:** Results of complementary log–log (discrete-time) and Cox proportional hazards (continuous-time) survival models of the co-primary outcomes.

Model	First co-primary outcome				Second co-primary outcome			
	Intention-to-treat		Per-protocol		Intention-to-treat		Per-protocol	
	HR (95% CI)	*p* value	HR (95% CI)	*p* value	HR (95% CI)	*p* value	HR (95% CI)	*p* value
Fixed effects only								
Time (univariable)	1.04 (1.03, 1.05)	<0.0001	1.04 (1.03, 1.05)	<0.0001	1.05 (1.03, 1.07)	<0.0001	1.05 (1.03, 1.06)	<0.0001
Intervention (univariable)	3.29 (1.79, 6.05)	<0.0001	3.19 (1.86, 5.47)	<0.0001	5.47 (2.39, 12.50)	<0.0001	4.85 (2.18, 10.79)	<0.0001
Intervention (adjusted for time)	1.42 (0.55, 3.72)	0.47	1.54 (0.76, 3.15)	0.23	2.60 (0.88, 7.63)	0.083	2.34 (0.86, 6.35)	0.096
Intervention (adjusted for age and time)	1.30 (0.48, 3.48)	0.60	1.41 (0.67, 2.98)	0.37	2.44 (0.81, 7.32)	0.11	2.20 (0.79, 6.11)	0.13
Intervention (Cox model)	1.60 (0.69, 3.71)	0.28	1.75 (0.77, 3.95)	0.18	NA	NA	NA	NA
Intervention (Cox model adjusted for age)	1.38 (0.59, 3.22)	0.45	1.52 (0.67, 3.44)	0.32	NA	NA	NA	NA
Mixed effects								
Intervention (adjusted for time)	1.40 (0.56, 3.51)∗	0.47	1.53 (0.64, 3.65)	0.34	2.57 (1.11, 5.96)	0.027	2.34 (1.05, 5.25)	0.039
Intervention (adjusted for age and time)	1.29 (0.51, 3.29)	0.59	1.43 (0.59, 3.47)	0.43	DNC	DNC	DNC	DNC
Intervention (Cox model)	DNC	DNC	1.75 (0.77, 3.95)	0.18	NA	NA	NA	NA
Intervention (Cox model adjusted for age)	1.39 (0.57, 3.40)	0.47	1.55 (0.65, 3.68)	0.32	NA	NA	NA	NA

Note: Unless otherwise indicated, the results of complementary log–log models are shown. Age-adjusted models are adjusted for age of participant at baseline. Discrete-time models were adjusted for time since the start of the trial in months, while time was specified in weeks for the continuous-time models Fixed effects models were fit with cluster-robust standard errors. A random effect could be fit for participant only in the model marked with an asterisk.

NA = not applicable; DNC = model did not converge; HR = hazard ratio.

### Per-protocol analysis

Results were similar to the intention-to-treat analysis. Over a median (IQR) of 42 (18) months of follow-up, 60 people experienced the first co-primary outcome during the trial, and 91 people experienced the second co-primary outcome. After adjustment for time, there was no evidence for an effect of the intervention on the first co-primary outcome (HR = 1.53; 95% CI 0.64, 3.65; *p* = 0.34). Results for continuous-time survival models were similar (Table 2). However, the intervention led to an increase in the second co-primary outcome (HR = 2.34; 95% CI 1.05, 5.25; *p* = 0.039).

## Discussion

Following implementation of the Let’s CHAT Dementia model of care, the rate of documentation of CIND/dementia diagnoses did not increase. However, the intervention did increase diagnostic processes used for assessment of dementia, with clients more than twice as likely to receive the best-practice diagnostic pathway during the intervention phase of the trial.

Several factors may have contributed to the lack of effect of the intervention on the first co-primary outcome. As described, multiple disruptions to the implementation program occurred, notably the start of the COVID-19 pandemic in early 2020, just as the Group 2 ACCHSs were entering the intervention phase. The project was hindered by extensive periods of lockdowns, coupled with restrictions being placed on research projects while ACCHSs operated in altered/emergency mode, and later, turned their focus to vaccination drives within their local communities. While requisite program adaptations, such as transitioning to online workshop delivery, was preferable to no workshops being delivered, facilitators experienced online delivery to be less impactful compared to in-person sessions. The in-person sessions had often combined other implementation or data collection activities and afforded research personnel the opportunity to spend time building relationships with ACCHS staff, an essential component of research with First Nations communities.^26^ The delay in delivering education and the concentration of workshops at the end of the implementation period means that potentially some of the effects of the intervention were not captured as they would have fallen outside the data collection period.

The diagnostic pathway process that occurs once CIND/dementia is suspected can be prolonged due to the multiple factors that need to be addressed to meet criteria for diagnostic certainty. This often includes complex referral systems^27^ and reluctance of the client and carers to have further investigation.^12^ This may account for the lack of an effect of the intervention on the documentation of CIND/dementia. To explore post-intervention measurement of co-primary outcomes, an additional “follow-up audit” at participating ACCHSs will track the 12-month period immediately following the implementation (underway at time of writing).

Documentation of CT and MRI brain scan referrals increased throughout the study period, potentially indicating a change in diagnostic practice, although the reasons for tests being requested was not always documented. This was facilitated by access to in-house geriatrician services in several ACCHSs (either pre-existing or implemented as part of the study). In line with the Royal Australasian College of Physicians’ Medical Specialist Access Framework,^28^ the in-house specialist model strengthens integrated, multidisciplinary care, is person-centred and family orientated, and enacts culturally safe care for First Nations clients by enabling care delivery in the familiar and community-based ACCHS setting. The success of the current study lends support to the expansion of this type of service, addressing the inequitable access to specialist services experienced by First Nations peoples and encouraging ACCHSs to extend their reach into areas of unmet need.

This study had several limitations. In terms of design, it may have been beneficial to include a transition period between the control and intervention phases, as done in some SWCRT studies.^21^ It generally took several months for the intervention to get underway in individual ACCHSs. Including these initial months where processes were being put in place but the intervention was not actually underway likely impacted the final outcomes. Further, as intervention delivery can change or be refined over time, timing and length of implementation is sometimes examined in SWCRTs.^21^ We were unable to do this due to timeline modifications resulting from COVID-19 disruptions. Loss of one ACCHS may have introduced selection bias into the per-protocol analysis, and the way we handled missing data in the continuous-time survival analyses may have introduced further bias. Additionally, our models do not account for the efficacy of the intervention potentially changing over time, the possibility of time-varying confounding, or bias introduced by competing risks. We also could not fully adjust for the effect of time during the trial, as participants would have experienced cognitive ageing throughout the study period.

We note also potential ‘rising tide effects’ and contamination possibilities for our study. For example, some of the co-researching ACCHSs were concurrently involved in other brain health initiatives or research while participating in the Let’s CHAT Dementia program. This could have had a positive or deleterious effect on the study outcomes depending on whether the additional programs were being undertaken during the control or intervention period. In addition, a national review of annual health checks (MBS item 715 and related items) during the study period resulted in updated recommendations for older people that introduced specific prompts regarding cognition.^29^ However, in our experience, many services were not aware of the updated recommendations. We made considerable efforts to assist with updating health check templates, however by the end of the study half the ACCHSs had not been able to incorporate the changes into their clinical software (evident during auditing).

In relation to First Nations-specific models of care, a systematic review of best practice models of aged care implemented for First Nations people identified the importance of a decolonising approach.^30^ Co-design processes that privilege the voices and care priorities of First Nations communities enable culturally safe and appropriate service delivery.^30^ The present study’s co-design approach took time to establish. We built relationships and sought input from ACCHS partners, tailoring the implementation to individual ACCHSs’ needs and preferences wherever possible. A recent systematic review on optimising the diagnosis and management of dementia within primary care reaffirmed that family physicians participating in educational seminars led to increased identification of dementia, and improved case management and outcomes for clients living with dementia and their caregivers.^31^ In the current study, ACCHS staff education was a major component of the intervention and involved both GPs and any staff member the ACCHS wanted to involve in the training. It also provided capacity-building opportunities for Aboriginal project officers and in many cases led to ongoing roles in ACCHSs or the research team. Case management models fit well with the multidisciplinary case management approach adopted by ACCHSs.

To conclude, the Let’s CHAT Dementia model of care intervention improved uptake of the diagnostic pathway for dementia amongst primary health care teams in the ACCHS context. This occurred despite ACCHS partners being substantially affected by the COVID-19 pandemic. These findings reinforce the appropriateness and importance of research and resources developed through co-design that incorporate cultural principles and content to improve dementia care outcomes for First Nations peoples. Given the diverse nature of the communities and services across different states of Australia as well as metropolitan, regional and remote settings, the generalisability of the trial findings to the First Nations community at large are promising. A process evaluation incorporating an Indigenous cultural lens was concurrently undertaken as part of this study and provides further insight into the mechanisms at play during the intervention.^32^ In addition, translational work is underway involving stakeholders in the broader sector with the aim of refining the model of care and embedding it into health professional educational curricula, policy and everyday practice.

## Contributors

DLG (project lead), LF, ST, KSm, ES, DA, DB, KR, MW, IB and SRu conceived the study and acquired the funding. DLG and RS developed the initial study design. Statistician ZH performed the statistical analyses and created data tables and charts. JH administered and supervised the project, with RS contributed to the development of the study (protocol development and ethics applications) and with MB and KB, study resources and procedures. RQ, SRu, RM and WA were regional coordinators of the study. LF, KSm, DB, KR, ES and DA were investigators on the project management group involved in an advisory capacity in all aspects of the trial. JH, KB, RM, HD, SRi, KSu, LP, BA, BMG, RQ, SRu, DCJ, VW, WA, KR, JC, LL, BD, TH, KF and SW contributed to data collection. JH and KB conducted data extractions and assisted with data analysis. DB and HD co-chaired the Indigenous Reference Group. JH and ZH wrote the initial draft of the manuscript. All authors critically reviewed the manuscript, contributed to the content and approved the final version. JH, KB, ZH, LF and DLG directly accessed and verified the underlying data reported in the manuscript. All authors had full access to the primary outcomes data. JH had the final responsibility to submit for publication.

## Data sharing statement

Data from this study will not be made available to others as they were collected under strict agreement with participating ACCHSs. Our study protocol has been published.^10^ Participation agreements with health services can be provided upon request via the corresponding author.

## Declaration of interests

We declare no competing interests.

## Appendix ASupplementary data

Supplementary Figs. S1–S3

Bradley et al. 2020
