# Supplementary material for: Implementation of a best-practice model of care for cognitive impairment and dementia for first nations peoples attending primary care in Australia: a stepped-wedge cluster-randomised trial
Source: Lancet Reg Health West Pac. 2025 Apr 3;57:101529. doi: 10.1016/j.lanwpc.2025.101529 (PMC12002872; doi:10.1016/j.lanwpc.2025.101529)
Supplement: Supplementary Figs. S1–S3 [file mmc1.docx]

**Supplementary Figure 1.** Percentage of people with documented cognitive impairment (A) and appropriate management of cognitive impairment (B) over the course of the trial, by health service

**
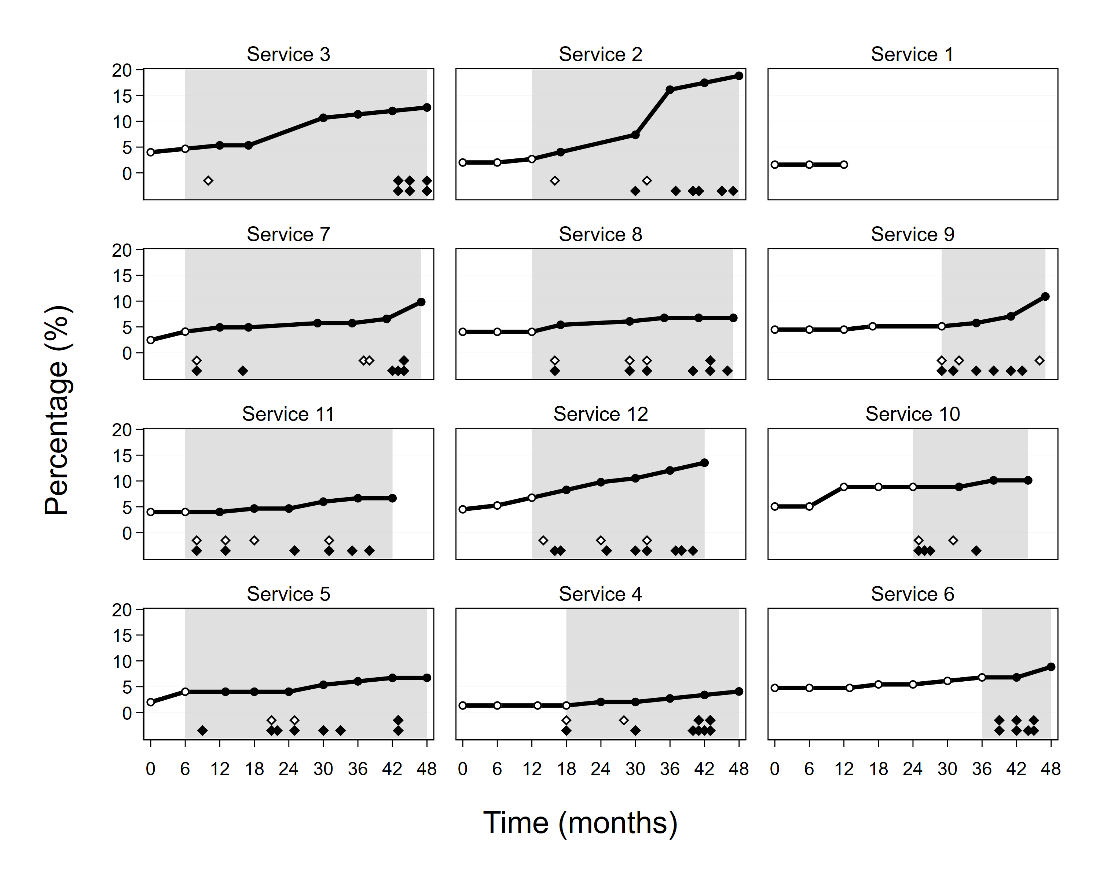

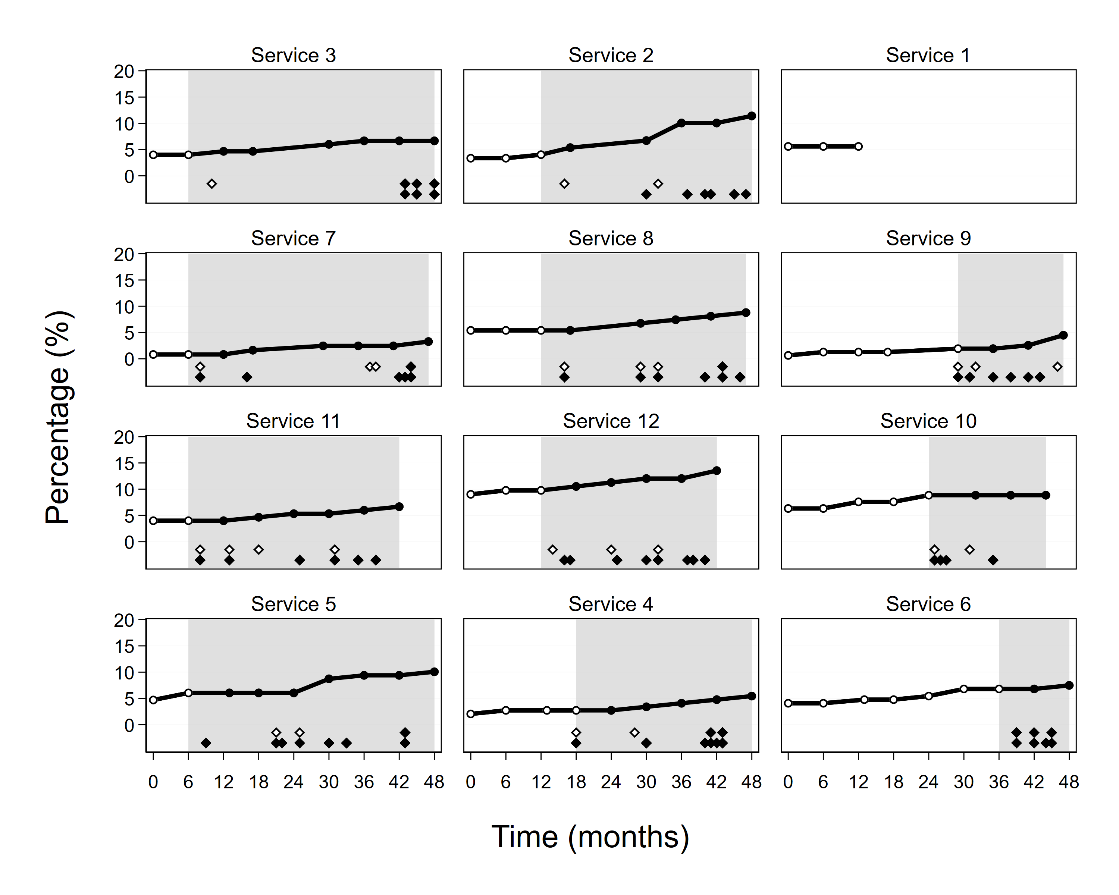
**

B.

A.

Note: Hollow circles denote control audits, while filled circles denote intervention audits. Shaded areas denote intervention period. Diamonds indicate when workshops took place (hollow diamonds depict general practitioner workshops). Individual workshops were repeated at some ACCHSs.

**Supplementary Figure 2**. Study flow diagram

**
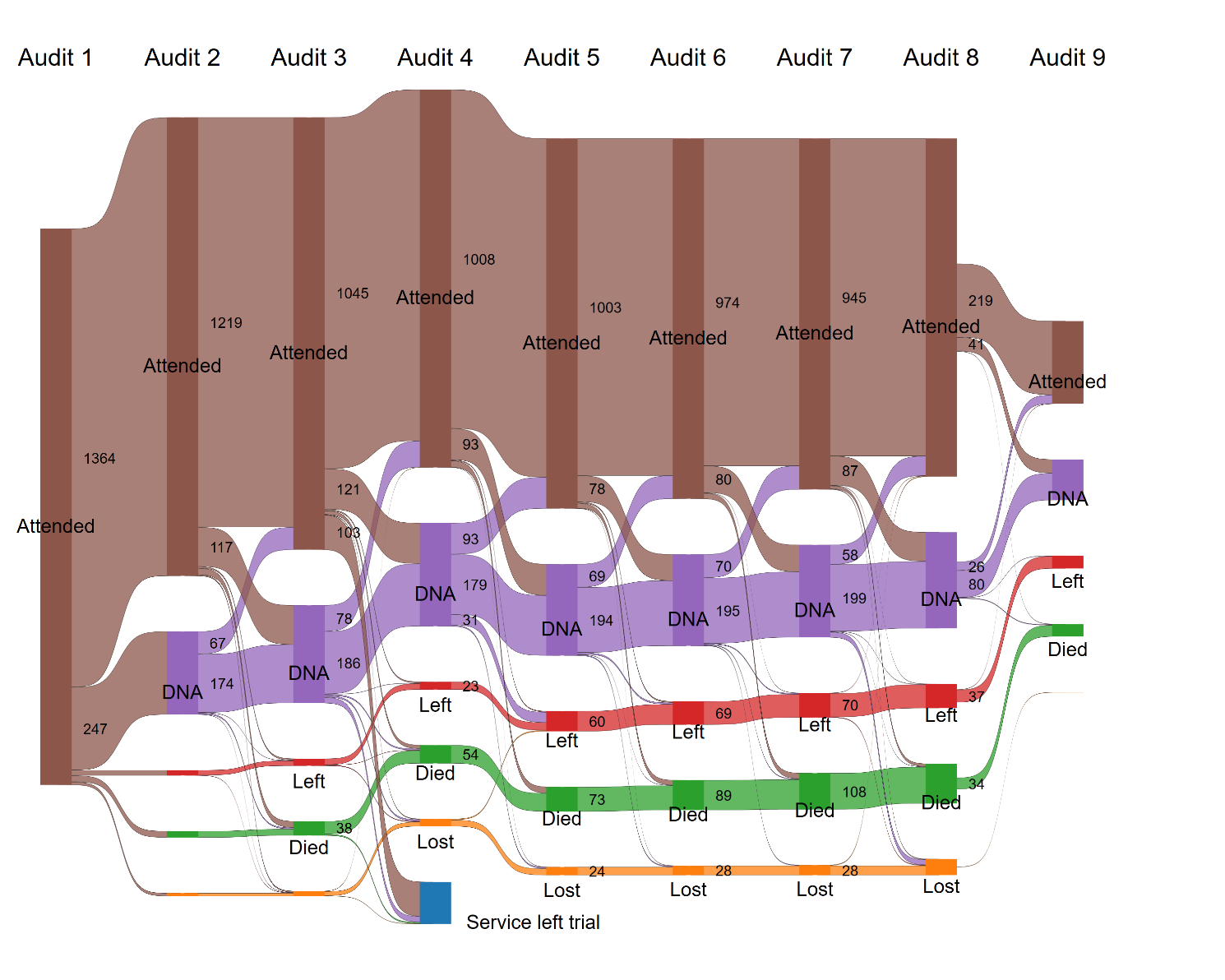
**

Note: Values less than 20 not shown. DNA = did not attend; left = left clinic; lost = lost to follow-up.

**Supplementary Figure 3**. Number and percentage of people with documented cognitive impairment (A) and evidence of uptake of appropriate diagnostic pathway (B) over the course of the trial

| **A**  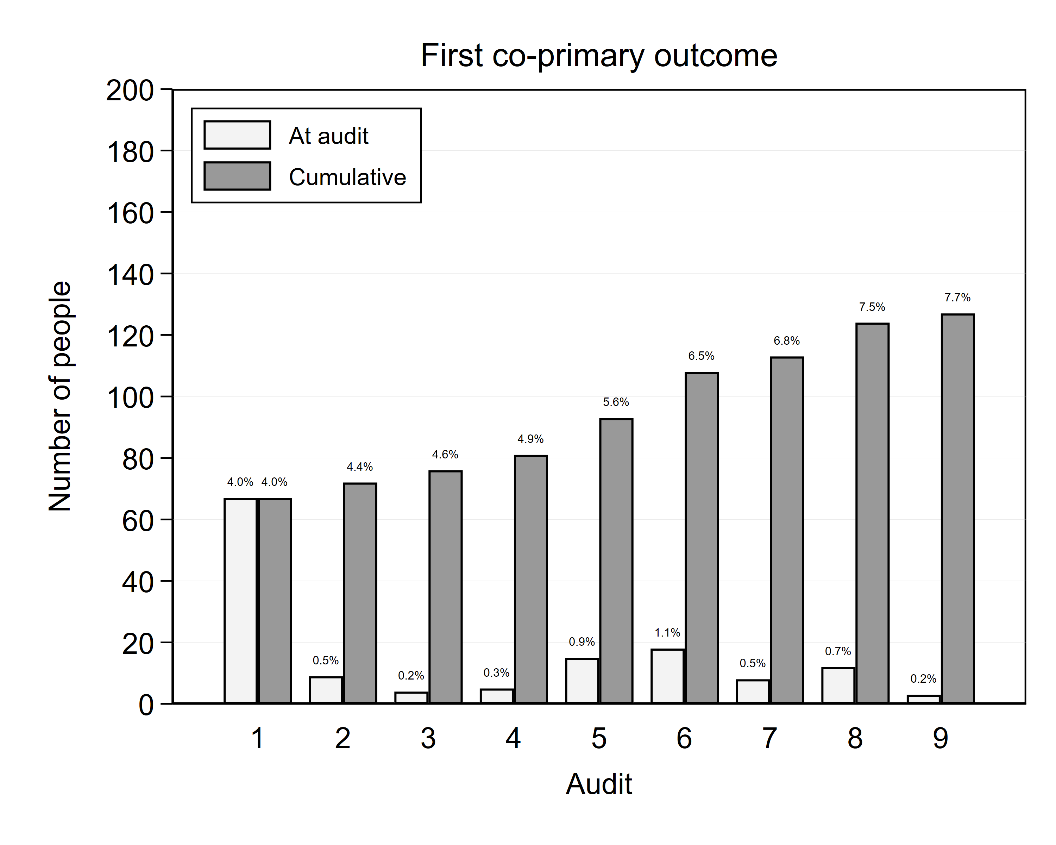 | **B**  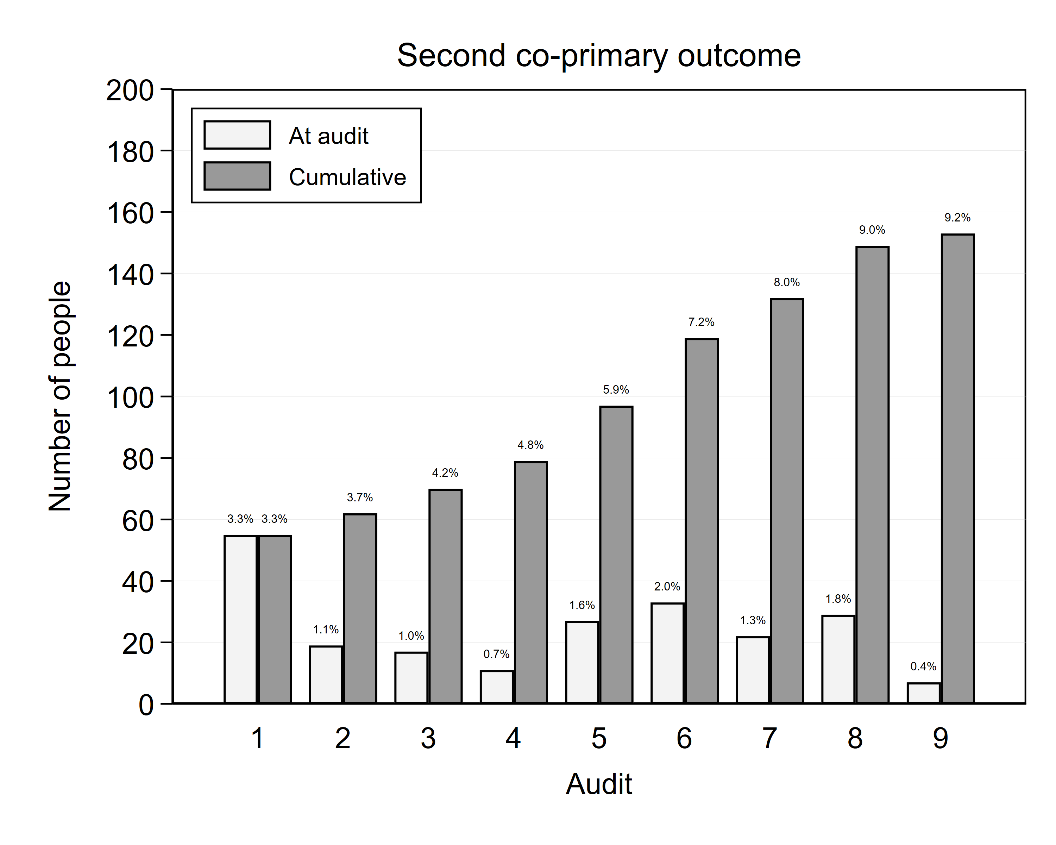 |
| --- | --- |
