# Supplementary material for: Implementation of a best-practice model of care for cognitive impairment and dementia for first nations peoples attending primary care in Australia: a stepped-wedge cluster-randomised trial
Source: Lancet Reg Health West Pac. 2025 Apr 3;57:101529. doi: 10.1016/j.lanwpc.2025.101529 (PMC12002872; doi:10.1016/j.lanwpc.2025.101529)
Supplement: Bradley et al. 2020 [file mmc2.pdf]

Bradley et al. BMC Health Services Research (2020) 20:208
https://doi.org/10.1186/s12913-020-4985-1

BMC Health Services Research

STUDY PROTOCOL

Open Access

Let's CHAT (community health approaches to) dementia in Aboriginal and Torres Strait Islander communities: protocol for a stepped wedge cluster randomised controlled trial

Kate Bradley[1*], Robyn Smith[1], Jo-anne Hughson[1], David Atkinson[2], Dawn Bessarab[3], Leon Flicker[2], Kylie Radford[4,5], Kate Smith[6], Edward Strivens[7], Sandra Thompson[8], Irene Blackberry[9] and Dina LoGiudice[1]

Abstract

Background: Documented rates of dementia and cognitive impairment not dementia (CIND) in older Aboriginal and Torres Strait Islander Peoples is 3–5 times higher than the rest of the population, and current evidence suggests this condition is under-diagnosed and under-managed in a clinical primary care setting. This study aims to implement and evaluate a culturally responsive best practice model of care to optimise the detection and management of people with cognitive impairment and/or dementia, and to improve the quality of life of carers and older Aboriginal and Torres Islander Peoples with cognitive impairment.

Methods/design: The prospective study will use a stepped-wedge cluster randomised controlled trial design working with 12 Aboriginal Community Controlled Health Services (ACCHSs) across four states of Australia. Utilising a co-design approach, health system adaptations will be implemented including (i) development of a best practice guide for cognitive impairment and dementia in Aboriginal and Torres Strait Islander communities (ii) education programs for health professionals supported by local champions and (iii) development of decision support systems for local medical software. In addition, the study will utilise a knowledge translation framework, the Integrated Promoting Action on Research Implementation in Health Services (iPARIHS) Framework, to promote long-term sustainable practice change. Process evaluation will also be undertaken to measure the quality, fidelity and contextual influences on the outcomes of the implementation.

The primary outcome measures will be rates of documentation of dementia and CIND, and evidence of improved management of dementia and CIND among older Indigenous peoples attending Aboriginal and Torres Strait Islander primary care services through health system changes. The secondary outcomes will be improvements to the quality of life of older Indigenous peoples with dementia and CIND, as well as that of their carers and families.

Discussion: The Let's CHAT Dementia project will co-design, implement and evaluate a culturally responsive best practice model of care embedded within current Indigenous primary health care. The best practice model of care has the potential to optimise the timely detection (especially in the early stages) and improve the ongoing management of people with dementia or cognitive impairment.

(Continued on next page)

* Correspondence: kate.bradley@unimelb.edu.au
[1]The University of Melbourne, Faculty of Medicine, Dentistry and Health Sciences, Royal Melbourne Hospital, Royal Park Campus, Administration Building 21, 34 -54 Poplar Road, Melbourne, Victoria 3052, Australia
Full list of author information is available at the end of the article

© The Author(s). 2020 **Open Access** This article is distributed under the terms of the Creative Commons Attribution 4.0 International License (http://creativecommons.org/licenses/by/4.0/), which permits unrestricted use, distribution, and reproduction in any medium, provided you give appropriate credit to the original author(s) and the source, provide a link to the Creative Commons license, and indicate if changes were made. The Creative Commons Public Domain Dedication waiver (http://creativecommons.org/publicdomain/zero/1.0/) applies to the data made available in this article, unless otherwise stated.

(Continued from previous page)
Trial registration: ACTRN12618001485224. Date of registration: 04 of September 2019

Keywords: Dementia, Alzheimer's disease, Cognitive impairment not dementia, Aboriginal and Torres Strait islander, Aboriginal community controlled health services

Background

Introduction

The Australian population of Aboriginal and Torres Strait Islander peoples is growing, and the number of people aged over 65 years is projected to nearly double by 2026 [1]. Older Aboriginal and Torres Strait Islander Australians play a crucial role in the health of their communities, including as stewards of cultural rights and responsibilities for maintaining connections to Country, caring for extended family members, and providing leadership and support within their families and within communities [2]. Although there are many examples of Aboriginal and Torres Strait Islander Elders 'ageing well' [3], research has documented a high prevalence and incidence of dementia and cognitive impairment not dementia (CIND) in Aboriginal and Torres Strait Islander Australians living in urban, rural and remote regions of Australia [4–6]. Rates of dementia and CIND are up to 5 times those observed in the non-Indigenous population, with onset at younger ages, and are influenced by potentially modifiable risk factors such as head injury, cardiovascular disease and stroke. This is in contrast with other research internationally, that demonstrates a decrease in dementia rates in some developed nations in the last three decades [7, 8].

There is increasing evidence that prevention and management of vascular and other risk factors could delay progression of dementia [9], and this may be of particular relevance in Aboriginal and Torres Strait Islander communities where there are high rates of co-morbid conditions [10, 11] and onset at earlier ages, including past head injury – a key risk factor associated with decline from normal cognition to impairment [4], and other associations including age, stroke, non-aspirin analgesics, lower BMI, and higher systolic blood pressure (BP) [4]. In addition childhood trauma was found to be a contributor to cognitive decline in urban regions [6, 12].

Primary health care teams play an essential role in the detection and management of dementia in the community, yet studies demonstrate that several factors affect poor detection of dementia in primary care. These include lack of confidence to diagnose dementia, therapeutic nihilism and lack of access to specialist services [13, 14]. In our study in the Kimberley, only 38% of those with dementia and 3% with CIND had been diagnosed by general practitioners (GPs) or had their diagnoses documented despite the study team informing the GPs of these diagnoses (personal communication by DL). This may reflect generally poor use of screening for early detection and management of chronic disease [15], with only 33% subsidised primary care screening completed for Aboriginal and Torres Strait Islander clients [15, 16]. Other factors affecting timely diagnosis of dementia for Aboriginal and Torres Strait Islander peoples include stigma, poor health literacy, perceived lack of culturally specific services, cultural understandings of dementia, fear of having to leave Country, financial costs and many competing priorities within family and communities [2, 3, 17] – factors affecting the timely diagnosis of all chronic conditions.

Evidence shows that GP education may increase the detection of suspected cases of dementia [18, 19] with resulting improved quality of life of older people [13], but effective translation also requires changes to routine systems. Previous successful primary care interventions to improve detection of dementia and CIND have included a combination of strategies: decision support systems, availability of guidelines, one-to-one and group education, and collaborative models of care including nurse-led interventions [18, 20]. Efficacy of primary care interventions for Aboriginal and Torres Strait Islander peoples can be very high, as demonstrated by studies such as the Aboriginal and Torres Strait Islander Health Worker led intervention for diabetes management [21] and a smoking intervention study in the Kimberley [22]. These and other studies emphasised the importance of cultural safety, utilising Aboriginal and Torres Strait Islander researchers and health workers, and a need for local health services to assume ownership of the project [20, 22]. Effective programs are also often supported by a framework of Continuous Quality Improvement (CQI) within Aboriginal and Torres Strait Islander primary care settings [23–25].

To date no studies have been undertaken locally or internationally to address the needs of Aboriginal and Torres Strait Islander people with dementia and CIND through primary care, despite the alarmingly high rates of these conditions. The Let's CHAT (Community Health Approaches To) Dementia program addresses the need to improve detection and management of dementia and CIND in primary care by using a holistic 'real world approach', based on adapting current systems and working collaboratively within ACCHSs and Communities to enable immediate translational outcomes.

The program will co-design, implement and evaluate a culturally responsive best practice model of care embedded within current Aboriginal and Torres Strait Islander primary health care and ACCHS systems and services. This will optimise the timely detection (especially in the early stages) and ongoing management of people with dementia or its precursor CIND.

Aims, objectives and outcomes of the study

The primary aim is to:

(i) Improve detection and management of dementia and CIND among older Aboriginal and Torres Strait Islander peoples attending primary care services through health system changes that include the following features:

(a) Collaboratively developed education programs delivered for capability building of health practitioners including Aboriginal and Torres Strait Islander Health Workers, nurses and GPs. This program will be supported by local champions.
(b) Development of a culturally appropriate Aboriginal and Torres Strait Islander best practice guide (BPG) for dementia and CIND, and;
(c) Decision support systems including software modifications and adaptations of the current Aboriginal Health Check (Medical Benefit Scheme (MBS) 715) templates;

The secondary aims include improvement in the quality of life of carers and older people with dementia and CIND.

Hypotheses

Primary hypothesis: Implementation of the Let's CHAT Dementia model of care and health system changes will result in an increase in documentation of cognitive assessment and management for dementia and CIND in the clinic population aged 50 and over.

Secondary hypothesis: Compared with usual care, implementation of the Let's CHAT Dementia model of care will: a) improve carers' quality of life; and b) improve health outcomes and quality of life of those with CIND and dementia and help with health economic evaluation.

Methods

Design

This research project has adopted a stepped wedge cluster randomised controlled trial design with 12 clusters (ACCHSs) across four states of Australia. This method was chosen for pragmatic and ethical reasons - each cluster will experience the intervention over the course of the study, contamination of the groups prior to intervention is minimised due to geographic separation, and the design minimises the difficulties of implementing a program simultaneously over large distances [26, 27].

In the context of Indigenous research, a community participatory approach is essential to ensure the community, service providers and stakeholders are involved at all stages enabling successful translation into sustainable outcomes [28]. The CQI "plan, study, do, act" cycle will be utilised in combination with the i-PARIHS framework with the purpose of being a practical and familiar way to engage staff in practice change [29, 30]. The clinical and support staff within each ACCHS will be invited by the research staff to co-develop and participate in the implementation phase of the project – consisting of staff education and resource development. Additionally, staff from each ACCHS and local Community members will be recruited to act as project Ageing Well Champions (AWC) – co-researchers and leaders for change [24]. The primary role of AWCs will be to assist with implementing the Let's CHAT Dementia model of care. They will be selected by ACCHS staff based on prior experience working with older Aboriginal and Torres Strait Islander Australian peoples. A protocol will be developed to outline the role of the champions.

A formal framework will be utilised to evaluate the nature and quality of the implementation, given the complexity of a multi-domain program and to determine barriers and enablers to care in a real-world health service intervention. In this project the iPARIHS framework has been selected to inform the knowledge translation aspects of the study [31]. This framework has been utilised for similar complex interventions in primary care and Aboriginal and Torres Strait Islander health care with successful outcomes [30]. iPARIHS adopts the following formula: $SI = Fac^{n}(I + R + C)$, where examination and understanding of the Innovation (what is being implemented and the evidence base supporting it), the Recipients (who is exposed to the implementation both individually and collectively) and the Context (inner and outer) are crucial in determining the Successful Implementation of an intervention. The approach positions the notion of Facilitation as the underlying element for the enablement of successful implementation. This framework will marry well with the co-design approach being taken in this study; facilitation will be a key area of focus to promote ongoing engagement from staff in co-developing and taking ownership of the iteratively designed model of care [30].

Setting

The study will be undertaken in North Queensland, New South Wales, Victoria and Western Australia with recruitment of three ACCHS in each state. Each health service (cluster) will be randomised to determine the

sequence of commencement of education and resource provision (the implementation phase) and the ACCHSs will enter the implementation phase of the study at six-monthly intervals. Randomisation will be carried out by a statistician external to the project team. One ACCHS per state will begin the implementation phase at a time. Allocation concealment will be ensured until the second group of ACCHSs enters the intervention phase, at which time all ACCHSs will be aware of their allocation sequence. Blinding of trial participants (ACCHSs) and data collectors will not be possible, since both groups will be aware of and possibly engaged in the implementation activities taking place once a health service enters the intervention phase. However, the research team plans to recruit external outcome assessors to check data samples. Table 1 (below) outlines the expected timeline for each phase of the project.

Participants/recruitment

Organisations

Twelve ACCHSs in total will participate in the study, three from each state involved. The recruitment of the health care services is ensured as there are existing good relationships with the practices, built during previous research and collaborative projects. The 12 ACCHSs will be initially engaged through conversations between senior management staff and project investigators, with facilitation by peak bodies in some states.

Individuals

Patients identified through the medical record audit as having documentation of suspected or confirmed dementia or cognitive impairment and their carers will be approached to participate in a comprehensive geriatric assessment (CGA). The individual assessments will serve the dual purpose of enabling the measurement of individual outcomes across the course of the study and feeding back care recommendations for patient management from project geriatricians to ACCHSs and assisting with health economic evaluation. For each person suspected/identified as having cognitive impairment or dementia two case controls matched for age and gender will also participate in the CGA wing of the study. A known staff member or clinician from the ACCHS will make contact, inform the potential participants about the study and introduce a research staff member. The research staff member will then speak with the person/family to provide further information on the study and invite the person to participate.

Inclusion criteria

The organisations [1] and individual participants [2] selected for the study will meet the following criteria:

1) ACCHSs that:
   (i) Utilise a health care software system with comprehensive electronic health records, such as MMEx or Communicare [32, 33];
   (ii) Utilise Medical Benefit Scheme (MBS) item 715 [34], which is a population-based health check specifically designed for health promotion, disease prevention and early diagnosis in Aboriginal and Torres Strait Islander populations;
   (iii) Have GPs and Aboriginal and Torres Strait Islander Health Workers, and/or practice nurses who assist in delivery of health checks;
   (iv) Participate in CQI [35]
   (v) Can provide at least 55 current Aboriginal and Torres Strait Islander patients who meet the inclusion criteria.

Table 1. Project timeline

	2017-8	Sept 2018-Aug 2019		Sept 2019-Aug 2020		Sept 2020-Aug 2021		Sept 2021-Jun 2023
Sites	0-12	12-18	18-24	24-30	24-36	36-42	42-48	49-66
1,2,3,4								Analysis, complete translation.
5,6,7,8								
9,10,11,12								

	Recruitment and training of Research Officers and Ageing Well Champions, development and testing of training tools and BPGs
	Audits, baseline assessments of people identified with CIND /dementia
	Implementation of Let's CHAT Dementia Model of Care, six-monthly audits

2) Aboriginal and Torres Strait Islander Peoples aged 50 years and over who are active clients (at least three visits in the last 2 years) of the health service. Despite the MBS 715 being available for all ages (with a category specific for 55 years and over), a 50-plus age group was chosen due to high rates of CIND and dementia documented in this group [36] and aligns with the age criteria for access to the Australian Government's aged care services platform, My Aged Care [37].

Exclusion criteria

Clients will be excluded from audits in the following cases. If they:

(i) Are severely unwell with high likelihood of death within 6 months;
(ii) Have not resided in the area for the last 12 months;
(iii) Currently reside in residential care and/or;
(iv) Are not active patients of the service as defined by the Royal Australian College of General Practitioners' definition: having attended no less than three visits to the service in the last 2 years [36].

Intervention

The implementation strategies for the intervention in each cluster will be determined in collaboration with the AWCs and local service leaders and will include the following elements:

(i) Education sessions, designed in collaboration with ACCHS staff including Aboriginal and Torres Strait Islander Health Workers, nurses and GPs, and provided to the ACCHS staff through a method that supports the needs of the health service. For example, workshops, webinars, online educational modules etc.; depending what the ACCHSs determine is the most effective form of training for their organisation.
(ii) Gaps and areas for practice development or learning will be identified with each health service team and organisation, based on results of the medical record audits developed and undertaken specifically for this project, together with feedback from each agency. The research team will engage with existing CQI processes in the health service to reinforce practice change behaviours.
(iii) An evidence-based best-practice guide to cognitive impairment and dementia care for older Aboriginal and Torres Strait Islander peoples in primary care will be developed and refined through extensive stakeholder consultation, including a modified Delphi process [38], workshops with the project's Translation Working Group, and advice from the project's Aboriginal and Torres Strait Islander Reference Group.
(iv) Adaptation and modification of the standard software used by primary care agencies to record and manage client care. The inclusion of prompts, decision support systems and adaptations to templates such as chronic disease care plans and Aboriginal Health Checks, and evidence-based guidance regarding dementia assessment and care will provide practical support for staff on a daily basis.
(v) Development of tailored care pathways for individual ACCHSs.

Outcomes

The *primary outcome* is new documentation of dementia or CIND within healthcare records. This includes: (i) evidence of cognitive screening, and/or (ii) documentation of inquiry into cognitive health (e.g. asking questions regarding memory; obtaining informant report regarding cognition; (iii) laboratory or imaging investigations specifically requested for assessment of dementia. The data informing the primary outcome measure will be collected during regular audit cycles (detailed below), both prior to and during the project implementation phase.

Secondary outcomes are focused on improved health and well-being of older Aboriginal and Torres Strait Islander peoples with dementia, their carers and families. This will be measured through two validated quality of life tools in the CGA (refer below).

Dissemination and translation

Formal feedback of outcomes to health services personnel will occur every 6 months. Regular community updates will be provided through various culturally appropriate media, including newsletters and posters. Two workshops will be held (at baseline and on completion of the project) in each region for consumers, carers, health professionals, aged care workers and all other relevant stakeholders to provide and discuss information, present findings, and to set research priorities for the future. Ongoing translation strategies will be developed in collaboration with AWC and relevant stakeholders.

Methods: data collection, management and analysis

Data collection and management

Medical record audit A specific audit tool has been developed to collect data from eligible ACCHS clients 50 years and over (see Table 2 for data collection timetable). The audit tool documents clients' dementia risk profile, any assessments or investigations relating to cognition, new diagnoses of dementia and CIND as well as describing the care pathways of any cognitively impaired clients, including referrals, hospitalisations and mortality. Data will

be collected by trained research assistants using REDCap; an online, secure, password protected, web-based data collection support tool. Data will be de-identified with a unique identifier code allocated to each participant to enable linking between successive audits, with data re-identifiable only in the clinical setting. Audits will be completed either by trained research assistants or ACCHS staff, depending on each service's preferences.

Comprehensive geriatric assessment (CGA) - client

Patients identified with possible dementia or CIND by audit and the gender and age matched case controls will be invited to be participants in the CGA. Different measures will be utilised in the CGA to gather data to assess individual outcomes throughout the life of the study. Cognition, quality of life, chronic pain, continence, daily function, mental health, history of psychosocial stress, nutrition, physical activity, sleep quality, frailty, visual and hearing impairment, muscle mass, blood pressure, grip strength and gait will be assessed (see summary table, Appendix 1, for standardised tools used). The CGA contains a detailed cognitive assessment section which includes the Kimberley Indigenous Cognitive Assessment, the Clock Test, Colour Trail Making Test and the Symbol Digit Modalities Test [39–42]. A semi-structured interview will be used to ascertain demographic factors, preferred language and education. Structured responses will be sought from the participants on the following: medical history, medications, past and present tobacco use (including chewing tobacco), past and present alcohol use, past and present drug use and history of head trauma. The CGA survey information will be collected by trained research assistants and will be entered and stored securely in REDCap. Paper copies will be stored in locked file cabinets in areas accessible by project research team members only.

Comprehensive geriatric assessment - Carers/ family

Carers and/or family will also be invited to complete a section of the CGA. They will be asked to assess the patient's health. Frailty, cognition, daily function, mental health, and nutrition will be assessed. In addition, a semi-structured interview will be used to ascertain both past and present smoking (including chewing tobacco), alcohol, and drug use by the patient from the carer and/or family member's perspective. Different measures will also be utilised to assess the carer's and/or family member's health, including a carer burden interview, a mental health assessment and a quality of life test. Individual and carer/family assessments will be completed by trained research assistants. The CGAs will be reviewed by geriatrician who will make care recommendations regarding clinical management and care which will be fed back to ACCHSs.

Data collection timetable

Table 2. Data collection timetable

Data collection	Baseline	6 monthly[a]	12 monthly[a]
Audit tool: demographic data & dementia mapping	X	X	X
Documentation of dementia or CIND (audit tool)	X	X	X
Audit documenting best practice	X	X	X
Participant: Comprehensive Geriatric Assessment	X		X
Carer: Outcomes	X		X
Health professionals: Process data	X	X	X

[a]6 monthly collection of data will continue from baseline to 36 months

Harms

Solicited and spontaneously reported adverse events and other unintended effects of the trial interventions or trail conduct will be managed by the Project Management team. This will be achieved through prior planning to mitigate potential harmful outcomes, and the availability of the Project Management team to address concerns and issues as they arise. For example, some of the questions in the CGA have the potential to cause distress to participants, as some questions ask the participants about possible negative life events they may have experienced. To mitigate and manage potential harmful outcomes for participants partaking in a CGA, researchers administering the CGA will be trained to follow a protocol for monitoring participant distress levels, as well as, reporting guidelines to follow in such cases. For example, dependant on the circumstances, it may be necessary for the researcher to consult with the participants GP who can then organise a referral to a psychologist, or perhaps, in a different circumstance, it may be appropriate to provide the participant with information about support services available in the local area.

Consent

Organisational consent will be sought from the ACCHSs for participation in the research project, including for the audits carried out as part of a quality assurance process. Individual informed consent will be sought from ACCHS staff participating in the co-design and evaluation of the best-practice model of care. Individual informed consent will also be sought from the clients, carers and family members participating in the CGA, this will be achieved by providing study participants with a Plain Language Statement that clearly outlines the study processes (refer to Appendix 2 Plain Language Statement).

For individual consent processes, interpreters will be used as required. Some of the people invited to take part

in the project will have dementia or other forms of cognitive impairment. If, as determined by a trained clinician, a person does not have capacity to give consent for participation in the detailed assessment and record linkage, consent will be sought from the carer, next of kin or a statutory health attorney/guardian.

Project evaluation

Process evaluation

A mixed methods approach will be used for the process evaluation of the implementation [43]. Fidelity, dose and reach of the intervention will be measured and evaluated by a project-appointed sub-committee.

Qualitative data for the process evaluation will be collected from all sites during the study. Data collection will include:

- Interviews with ACCHS staff at multiple time points during the implementation phase of the study to capture experiences of the intervention. Interview participants will be recruited via purposive sampling, to provide a representative sample of different roles within the health service (eg. GPs, nurses, AHWs, administrative staff, allied health staff) levels of exposure to (and possibly attitudes towards) the implementation;
- Project research staff notes documenting observations made during visits to ACCHSs and other contact with ACCHS staff (including phone calls and emails);
- Any feedback from AWCs regarding the implementation.

The iPARIHS framework will be used to guide the implementation. As such, all data collected for the process evaluation will focus on the characteristics of the implementation of the best-practice model of care for cognitive impairment and dementia (the innovation), who is affected by it (the recipients) and how they are affected by it, the inner and outer contexts within which the model of care is being implemented, as well as the facilitation of the implementation.

Health economic analysis

An economic evaluation will be conducted as part of this study. Best-practice methods will be utilised to undertake a cost-utility analysis of the intervention with quality of life as measured and valued by the EQ-5D as the primary outcome measure [44]. Resource use associated with the development and implementation of the Let's CHAT model of care (including staff time spent developing and administering the program, consumables, software development and others) will be carefully documented, measured and valued.

Quality Assurance & Data Monitoring

A Data Monitoring Committee (DMC) has been established. The DMC is not independent from the study organisers and is composed of the central management team. The DMC will undertake check and data cleans periodically for all study sites to ensure data collection adheres to the study protocol. Spot checks will also be undertaken on a semi-regular basis by a member of the DMC to assess the reliability of the data collected from the different ACCHSs involved in the study. Quality assurance of data collected in this study will be validated through electronic checks via the REDCap system (used for data collection in this project). These data monitoring processes will ensure that the quality and completeness of the data will be maintained over the course of the project.

Analysis and sample size

Analysis

Statistical analysis will be conducted using generalised linear mixed models (GLMM) where variation between clusters will be modelled as a random intercept effect and, nested within these, time will be treated as a random coefficient effect. No single model can assess all the possible time-related effects that may be of interest and to this end up to four different model configurations will be used as outlined in Twisk et al. [45].

Sample size

Previous work in the Kimberley (confirmed by work in NSW) found that in Aboriginal and Torres Strait Islander communities, based on community screening, the true prevalence of dementia in people aged 45 years and over was approximately 10%, and the prevalence for cognitive impairment without dementia was also approximately 10% [5, 45]. Hence, the true prevalence of dementia or cognitive impairment in the relevant population is 20%. In the Kimberley, only 38% of those with dementia and only 3% of those with cognitive impairment had been diagnosed by primary health care practitioners. Moreover, the fraction of dementia cases identified by primary care in this work was likely to be higher than in standard practice as many of those with diagnosed dementia had been identified as part of previous research, with primary care practitioners subsequently notified. Therefore, we assume that no more than 15% of individuals with cognitive impairment or dementia are currently identified by primary health practitioners. The initial prevalence based on detection in primary care will be 15% of 20%, i.e. 3%. At a minimum, the study intervention should enable practitioners to identify at least half (50%) of the primary care patients with dementia or cognitive impairment. This would increase the prevalence based on detection in primary care to 50% of 20%, i.e. 10%. If an intraclass correlation of 0.01 and an average cluster size of 50 individuals is assumed, then the

design effect will be 1.49. If the study was an individually randomised trial and we wished to detect an increase in the identification of cognitive impairment and dementia from 3 to 10% then the sample size would need to be at least 388 people (α = 0.05, power = 0.8). Therefore, the sample size required for this cluster randomised study will be 578 (388 × 1.49). Allowing for 10% drop-outs we plan to recruit 12 clusters of at least 55 people each, a total sample size of at least 660 individuals. Secondary outcomes compare the effect of earlier detection on scales of depression and stress and this sample size also has adequate power for these scales. We expect approximately 18 cases to be detected in the control period and 60 in the intervention period. The average cluster size will be 6.5 and assuming an ICC of 0.01, the design effect will be 1.055. Thus, our effective number of control cases will be 17 (18/1.055) and intervention cases 57 (60/1.055). Based on the variation found on the longitudinal data of carer strain in the Kimberley, our proposed study will provide over 80% power to find any clinically important effects.

Discussion

The Let's CHAT Dementia project aims to deliver a model of care for cognitive impairment and dementia in Aboriginal and Torres Strait Islander primary care settings. The co-design nature of this study will be essential to maximise learning and ensure a successful and sustainable result. Previous research has demonstrated the efficacy of participatory research design frameworks in Aboriginal and Torres Strait Islander health contexts. Such research projects have proven successful because they embrace two-way learning and are adapted to the cultural and historical community setting, increasing chances of uptake [28]. This project will involve the ACCHS research partners and their staff in decision-making processes pertaining to the design and implementation of the study, as well as appointing a project Aboriginal and Torres Strait Islander Reference Group whose members will be Aboriginal and Torres Strait Islander researchers, professionals in the health sector and community members with a background/interest in advocating for Elders, ageing well and cognitive health issues. Health care professionals are ideally positioned to help inform the design of interventions in their own work contexts. They have firsthand, experiential knowledge of the environment and the people within it, including specific cultural knowledge of the groups being served. Co-design frameworks also provide an important safeguard for communities involved in research, and are a key attribute in providing cultural safety to vulnerable groups [46]. Extensive, regular consultation with a range of Aboriginal and Torres Strait Islander representatives on all aspects of the project throughout the course of this research will help to ensure that the project is conducted ethically, appropriately and respectfully.

The prior translational work undertaken with older Aboriginal and Torres Strait Islander Australians appropriately positions this study toward reaching the project's main goals [2, 39, 47]. Such work includes development and validation of culturally appropriate screening tools, the development of health and community care pathways, and the detailed documentation of unmet needs of those with dementia and their carers, and, the previous successful implementation of primary care interventions in Aboriginal and Torres Strait Islander settings [2, 39, 47]. In addition, substantial groundwork in the form of developing networks and relationships with communities, services and stakeholders across the four states involved has already been laid.

A strength of this study is the adaptability of the implementation design to meet the needs of each specific health practice context (the individual ACCHSs). However, this may equally impact the ability of the project to maintain fidelity when dealing with the disparate groups involved. While this approach could affect the generalisability of conclusions drawn from the research, the relatively large number of sites involved (n = 12) from four states in Australia will provide the study with rich, qualitative data both in terms of individual case studies for each health service and aggregated service data concerning the processes undertaken. This means that, considered globally or in parts, the transferability of the research design and model of care of the study to other Aboriginal and Torres Strait Islander health contexts will be high.

Lastly, a potential barrier to this study will be the implementation of the CQI process within each health service, which is a central methodological pillar of this study with the purpose of facilitating reflective thinking in the ACCHS. Current international literature highlights that the implementation of CQI is difficult, the effects are variable and little evidence exists on the factors enhancing or impeding successful implementation of CQI [48]. Conversely, there is a growing body of literature which demonstrates the effectiveness and the importance of CQI frameworks in improving service delivery and clinical outcome measures for Aboriginal and Torres Strait Islander people and, further, CQI programs are widely accepted and implemented by ACCHSs in the Australian context [23, 48, 49]. It is hoped, through the utilisation of the CQI "plan, do, study, act" [29] cycle supplemented by audit tools developed to support actioning plans that are guided by best practice and developed alongside the i-PARIHS framework, barriers to the implementation of CQI will be overcome in this project [30].

Conclusion

During this project ACCHS staff will improve capacity to diagnose, manage and support older Aboriginal and Torres Strait Islander clients with cognitive health concerns, and

their carers, families and communities. Staff will have the opportunity to actively participate in research and share or further develop their skills. In the participating ACCHSs, best practice recommendations for cognitive impairment and dementia care will be provided along with review and quality improvement of services. These include support to achieve required reporting and strengthening of service development activities. This study will test ways in which to build staff capacity and foster the translation of best evidence into everyday best practice.

Considering the higher rates of dementia in the Aboriginal and Torres Strait Islander Australian population and the current evidence that dementia and CIND is underdiagnosed and under-managed in a clinical primary care setting, there is a need for research that addresses the need for improved detection and management of dementia and CIND among older Aboriginal and Torres Strait Islander peoples attending ACCHS and a focus on the improvement of quality of life for older Aboriginal and Torres Strait Islander peoples with dementia and cognitive impairment, their carers, families and Communities.

Although there is no cure for dementia, there are many modifiable risk factors that can be addressed across the life-course. Further, secondary prevention measures can be effective for those with cognitive impairment, and greater support and more effective management are possible once people have a diagnosis. Timely diagnosis gives the best opportunity to ensure that optimal care is provided and that each individual and their carer and family are well supported. Thus, strengthening the capacity of ACCHSs to detect and manage the dementia journey with older clients is a priority for health care research.

Appendix 1

Comprehensive geriatric assessment; standardised tool list

Appendix 1 *(Continued)*

Comprehensive Geriatric Assessment: List of Standardized Tools included.	Description of Standardized Tools.
Charlson Comorbidity Index (CCI) [50].	The CCI was developed to measure the comorbidity in relation to life expectancy [50]. For this project, the language utilized in this tool has been adapted to be more culturally appropriate.
Current Alcohol Consumption – Audit C Tool [51].	The AUDIT -C Tool is a three-question abbreviated version of the alcohol use disorders test (AUDIT). The tool is used to assess hazardous alcohol usage [51].
Kimberley Indigenous Cognitive Assessment (KICA) [39].	The KICA is a validated cognitive screening tool designed to assess cognition in older Indigenous Australians [39].
Colour Trail Making Test (CTMT) [41].	The CTMT was developed to assess sustained attention in adults, whilst being free of both language and cultural bias [41].
Digit Symbol Modalities Test (DSMT) [42]	The DSMT is designed to detect cognitive impairment by screening for organic and cerebral dysfunction [42].
EQ 5D [52].	EQ 5D is a comprehensive quality of life instrument developed to explain and value health [52].
Good Spirit Good Life Quality of Life Tool (GSGL) [53] .	The GSGL Tool was designed to identify the quality of life needs of older Aboriginal Australians with cognitive impairment and dementia [53].
Rapid Assessment of Physical Activity (RAPA) [54].	The RAPA was designed to assess levels of physical activity among adults 50 years and over [54].
Elderly Falls Screening Scale (EFST) [55].	The EFST is a five -Item test used to assess falls risk in the community-dwelling elderly [55].
Modified International Consultation Incontinence Questionnaire (ICIQ) [56].	The ICIQ is a questionnaire which was developed to assess urinary incontinence and its effect on quality of life [56]. For the purposes of this project the ICIQ has been modified to be more culturally acceptable.
Global Sleep Assessment Questionnaire (GSAQ) [57]	The GSAQ is an assessment tool developed to screen for sleep disorders [57].
Kimberley Indigenous Cognitive Assessment -Depression (KICA - Dep) [58].	The KICA – Dep was developed as a culturally sensitive and valid scale to assess depressive symptoms in older Indigenous Australians [58].
Exhaustion - CES–D Depression Scale [59].	Two questions were taken from The CES -D Scale and included in the comprehensive geriatric assessment to assess exhaustion. The CES – D Scale is a self- report scale to measure depressive symptoms [59].
Geriatric Anxiety Inventory - Short Form (GAI-SF) [60].	The GAI -SF is a 5-item version of the Geriatric Anxiety Inventory developed for measuring Generalized Anxiety Disorder [60].
Negative Life Events Scale (NLES) [61].	The NLES was developed to measure chronic stress in Indigenous Australian populations [61].
Mini Nutritional Assessment (MNA) [62].	MNA is validated assessment instrument developed to assess nutritional problems [62].
Fried Frailty Index (FFI) [63].	FFI is an index used to assess frailty in older adults [63].
Kimberley Indigenous Cognitive Assessment (KICA)- Carer [64].	The KICA Carer is an informant information questionnaire designed to supplement the Kimberley Indigenous Cognitive Assessment

Appendix 1 ***(Continued)***

Comprehensive Geriatric Assessment: List of Standardized Tools included.	Description of Standardized Tools.
	Tool [64].
Adapted Zarit – 6 item Carer Burden Interview [65].	The Burden Interview (ZBI) is a self-report measure to assess the level of burden experienced by caregivers [65]. For the purposes of this project the interview has been adapted from 29 item version to a 6 item version [66].

Appendix 2

Informed Consent Materials

Plain Language Statement.

Project: Let's CHAT (Community Health Approaches to) Dementia.

A/Prof Dina LoGiudice, Dr. Jo-anne Hughson (Responsible Researcher)Tel: + 61,481,900,008 Email: Dina.LoGiudice@mh.org.au, hughson@unimelb.edu.au

What is this research about?

This research project will work to improve health care for people in the [insert name here] Community aged 50 and over. We will do this by checking health records every 6 months, and working with the staff at the health service and with the community to look after older people better. We hope this will improve health and well-being for individuals, their families, carers and the Community. We are interested in thinking and memory problems that might be linked to dementia. Being part of this study doesn't necessarily mean you have dementia or memory issues.

What will I be asked to do?

One of our trained research assistants will ask you questions about your memory and thinking, and do some health checks. This will take about 1 h, and a half and will happen every year, for 4 years. The research assistant can meet you at the clinic or at your home. S/he will also talk with your main carer or someone in your family to ask how they are and how much help they give you. This information will be kept confidential, or shared with your doctor and clinic if you agree.

We also want to learn about how much health care costs for people over 50 in Aboriginal Communities. This includes when you go to the doctor, the hospital, have tests or take medicines. Medicare, the Pharmaceutical Benefits Scheme (PBS) and the State Health departments keep records of this information for everyone. With your consent, we can access your information. A consent form is attached, which includes an example of the records we would receive. The consent form is sent securely to the Department of Human Services and this information is kept private. We will also ask for access to records of hospital admissions, emergency departments, and ambulance services via the State Health departments. If you agree to this, we will handle all information carefully to make sure it is safe. We will then combine this information with the information we collect talking to you and your carer.

What are the possible benefits?

We hope that by training and supporting the staff of Health Services we can improve the care and health of older Aboriginal and Torres Strait Islander peoples, their families and community. To help cover your time or any out of pocket costs, we can reimburse you $50 each time you are interviewed.

What are the possible risks?

There is a low risk that people may be upset by some questions asked in the interviews. You can stop the interview at any time. If you feel upset by any questions, the researcher will help you get support from the health service.

Do I have to take part and what happens to my information?

Being part of the study is completely voluntary. You can pull out at any time. If you wish to pull out, you can do so by contacting [nominate an appropriate "arms length" person/local contact for each jurisdiction/ACCHS and give phone number]. If you decide to pull out, we will not collect additional personal or health information from you, but if you give us permission we will use the information you have already provided.

We are very careful about protecting your privacy. We use number codes instead of names to store most of your information, so that only the people working directly with you will know your name and contact details. Researchers who look at your results later will only see the number codes, not your name and nothing that identifies you individually will ever be reported on.

Paper-based consent forms will be stored in a locked cabinet in the project team's office at the Royal Melbourne Hospital. Computer files will be stored without identifying information such as names or date of birth, on a password protected research study computer which only the research team can access. By law, we need to keep the research data for 5 years after the study is finished. After this period, it will be destroyed.

Who is funding this project?

This project is funded by the National Health and Medical Research Council (NHMRC).

Where can I get further information?

If you would like more information about the project, please contact the researchers; A/Prof Dina LoGiudice or Dr. Jo-anne Hughson (Responsible Researcher).

Tel: + 61,481,900,008 Email: Dina.LoGiudice@mh.org.au; hughson@unimelb.edu.au

Who can I contact if I have any concerns about the project?

Your health service is working with the research team on this project. This project has been approved by the

Aboriginal Health and Medical Research Council of NSW and the Western Australian Aboriginal Health Ethics Committee (WAAHEC). If you have any concerns or complaints about the conduct of this research project you can talk to [local health service coordinator]. If you do not wish to talks to the research team or the health service, you can contact: The Chairperson, AH&MRC Ethics Committee, Tel: (02) 9212 4777 or The WAAHEC Ethics Officer, Email: ethics@ahcwa.org, Tel: (08) 9227 1631.

All complaints will be treated confidentially. In any correspondence please provide the name of the research team or the name or HREC reference number of the research project.

Abbreviations

ACCHSs: Aboriginal community controlled health services; AWC: Ageing well champion; BP: Blood pressure; CIND: Cognitive impairment not dementia; EQ-5D: Quality of life questionnaire; GPs: General practitioners; KICA-Cog: Kimberley indigenous cognitive assessment; MBS: Medicare benefits scheme; NHMRC: National health and medical research council; NSAF: National screening assessment; PARiHS: Promoting action-on research implementation in health services); PBS: Pharmaceuticals benefit schem; PMG: Project management group

Acknowledgements

We would like to acknowledge the ACCHSs who are taking part in the study: Wathaurong Aboriginal Co-operative, Ballarat and District Aboriginal Co-operative, Mallee District Aboriginal Services, Bidyadanga Aboriginal Community La Grange Inc. Health Centre, Broome Regional Aboriginal Medical Service, Derbarl Yerrigan Health Service Aboriginal Corporation, Durri Aboriginal Corporation Medical Service, Galambila Aboriginal Health Service, Tharawal Aboriginal Corporation Aboriginal Medical Service, Gidgee Healing, Mamu Health Service Limited and Wuchopperen Health Service Limited.

Trial results

The results of this trial will be disseminated to the trial participants, health care professionals, the public and the ACCHSs involved in the project. The audit results and the results from CGAs will be fed back to local services as formal feedback outcomes – each ACCHSs will receive only the data specific to their service.
Additionally, the audit and CGA results will also be disseminated through publications and reports accessible to the public and healthcare professionals – all published data will be in aggregate form and will be deidentified to ensure confidentiality. Results for this trial will be disseminated regardless of the magnitude or direction of effect.

Reproducible research

The full protocol is being made available in full through journal publication.

Protocol amendments

Any modifications to the protocol which impact on the study design or conduct will require a formal amendment to the protocol. Such an amendment will be agreed upon by the Project Management Group (PMG) and will be need to be approved by the following ethics committees; The University of Melbourne, Human Research Ethics, Western Australian Aboriginal Health Ethics Committee, Aboriginal Health and Medical Research Council, Human Research Ethics Committee.
Administrative changes of the protocol which are minor corrections and/or clarifications that have no effect on how the study is conducted will be agreed upon by the PMG, and will be documented.

Sponsor contact information

Trial Sponsor: The University of Melbourne.
Contact Name: Assoc Prof. Dina LoGiudice.
Address: The University of Melbourne, Faculty of Medicine, Dentistry and Health Sciences, Royal Melbourne Hospital, Royal Park Campus, Administration Building 21, 34–54 Poplar Road, Parkville Victoria 3052.

Committees

Organisational Structure and responsibilities.

Project Management Group:

- Agreement on the final protocol
- Membership: all chief investigators, the project manager, co-chairs of the Indigenous Reference Group, two representatives from the Translation/Model of Care Working Group. Other team members as required.
- Recruitment of ACCHSs and liaising with lead investigator
- Reviewing progress of study and if necessary agreeing on changes to the protocol
- Responsible for the ethical and appropriate conduct and governance of the project
- Responsible for the state-based working groups, Translation/Model of Care working group and liaison with the Indigenous Reference group.

Indigenous Reference Group:

- Membership: at least three people per project state (Vic, NSW, Qld, WA) with experience as advocates for Indigenous people, such as an Elder, carer of someone with dementia, care worker or community Member with a strong advocacy role.
- Provide expertise, guidance and advice to ensure the research project methods and outcomes are culturally appropriate, take account of specific local issues and are undertaken in ways that promote capacity building, particularly in local communities.

Translation/ Model of Care Working Group

- Ensure the project produces an effective model of care
- Advise on potential avenues for knowledge translation.

State-based Working Groups (one each in New South Wales, Queensland, Victoria and Western Australia)

- Responsible for the enactment of the project in the respective state.
- Reports to the Project Management Group

Authors' contributions

KB is the Research Assistant involved in drafting and finalising the manuscript. RS is a Project Consultant who participated in drafting and revising the manuscript critically for important intellectual content. JAH is the Let's CHAT Dementia Project Manager who participated in drafting, finalising and revising the manuscript for important intellectual content. DA is a Chief Investigator who participated in the study design and revising the manuscript critically for important intellectual content. DB is a Chief Investigator who participated in the study design and revising the manuscript for important intellectual content. LF is a Chief Investigator who participated in the study design and revising the manuscript for important intellectual content. KR a Chief Investigator who participated in the study design and revising the manuscript for important intellectual content. KS a Chief Investigator who participated in the study design and revising the manuscript for important intellectual content. ES is a Chief Investigator who participated in the study design and revising the manuscript for important intellectual content. ST is a Chief Investigator who participated in the study design and revising the manuscript for important intellectual content. MW is a Chief Investigator who participated in the study design and revising the manuscript for important intellectual content. IB is a Chief Investigator who participated in the study design and revising then manuscript for important intellectual content. DLoG is the Lead Investigator who conceived the study and participated in the design, finalising and revising the manuscript for important intellectual content.
All the aforementioned authors have read and approve the following manuscript to be published.

Funding

This project is funded by two National Health and Medical Research Council (NHMRC) grants: a Boosting Dementia Research grant (2017–2020) and a Targeted Call for Research in Indigenous Health grant (mid-2018-2023). The funder had no role in the design of this study and will not have any role during its execution, analyses, interpretation of the data, or decision to submit results.

Availability of data and materials
Currently, there is no data or materials available for publication.

Ethics approval and consent to participate
Currently this project has ethics approval from: The University of Melbourne, Human Research Ethics, Ethics ID 1851943; Western Australian Aboriginal Health Ethics Committee, reference number 858; Aboriginal Health and Medical Research Council, Human Research Ethics Committee reference number 1362/18.
Written consent will be obtained for this study at both the organisational and individual level.

Consent for publication
There is consent from all authors for this paper to be published.

Competing interests
The authors declare that they have no competing interests.

Author details
[1]The University of Melbourne, Faculty of Medicine, Dentistry and Health Sciences, Royal Melbourne Hospital, Royal Park Campus, Administration Building 21, 34 -54 Poplar Road, Melbourne, Victoria 3052, Australia. [2]The University of Western Australia, Rural Clinical School of Western Australia , PO Box 1377, Broome 6725, Australia. [3]The University of Western Australia, M303, 35 Stirling Highway, Perth 6009, Australia. [4]Neuroscience Research Australia, 139 Barker Street, Sydney, NSW 2031, Australia. [5]The School of Medical Sciences, University of New South Wales, Sydney, NSW, Australia. [6]University of Western Australia, 35 Stirling Highway, Perth 6009, Australia. [7]Queensland Government, Brisbane, Australia. [8]The University of Western Australia, 167 Fitzgerald St, Geraldton, WA 6530, Australia. [9]LaTrobe University, PO box 821, Wodonga, VIC 3689, Australia.

Received: 12 November 2019 Accepted: 12 February 2020
Published online: 12 March 2020

References
1. Australian Bureau of Statistics (ABS). ABS Estimates and Projections, Aboriginal and Torres Strait Islander Australians, 2001 to 2016. Canberra; 2014.
2. Smith K, Flicker L, Shadforth G, Carroll E, Ralph N, Atkinson D, et al. "Gotta be sit down and worked out together": views of Aboriginal caregivers and service providers on ways to improve dementia care for Aboriginal Australians. Rural Remote Health. 2011;11(2):1650.
3. Arkles R, Jackson Pulver L, Robertson H, Draper B, Chalkley S, Broe A. Ageing, cognition and dementia in Australian aboriginal and Torres Strait islander peoples: a life cycle approach; 2010.
4. Lo Giudice D, Smith K, Fenner S, Hyde Z, Atkinson D, Skeaf L, et al. Incidence and predictors of cognitive impairment and dementia in aboriginal Australians: a follow-up study of 5 years. Alzheimers Dement J Alzheimers Assoc. 2016;12(3):252–61.
5. Radford K, Mack HA, Draper B, Chalkley S, Daylight G, Cumming R, et al. Prevalence of dementia in urban and regional aboriginal Australians. Alzheimers Dement. 2015;11(3):271–9.
6. Li SQ, Guthridge SL, Aratchige PE, Lowe MP, Wang Z, Zhao Y, et al. Dementia prevalence and incidence among the indigenous and non-indigenous populations of the Northern Territory. Med J Aust. 2014;200(8):465–9.
7. Matthews FE, Arthur A, Barnes LE, Bond J, Jagger C, Robinson L, et al. A two-decade comparison of prevalence of dementia in individuals aged 65 years and older from three geographical areas of England: results of the cognitive function and ageing study I and II. Lancet Lond Engl. 2013;382(9902):1405–12.
8. Satizabal CL, Beiser AS, Chouraki V, Chêne G, Dufouil C, Seshadri S. Incidence of dementia over three decades in the Framingham heart study. N Engl J Med. 2016;374(6):523–32.
9. Baumgart M, Snyder HM, Carrillo MC, Fazio S, Kim H, Johns H. Summary of the evidence on modifiable risk factors for cognitive decline and dementia: a population-based perspective. Alzheimers Dement. 2015;11(6):718–26.
10. Australian Institute of Health and Welfare. The health and welfare of Australia's Aboriginal and Torres Strait Islander people, an overview 2011.
11. Hussain MA, Katzenellenbogen JM, Sanfilippo FM, Murray K, Thompson SC. Complexity in disease management: a linked data analysis of multimorbidity in aboriginal and non-aboriginal patients hospitalised with atherothrombotic disease in Western Australia. PLoS One. 2018;13(8):e0201496.
12. Radford K, Lavrencic LM, Delbaere K, Draper B, Cumming R, Daylight G, et al. Factors Associated with the High Prevalence of Dementia in Older Aboriginal Australians. J Alzheimers Dis. 2018;Preprint(Preprint):1–11.
13. Mate KE, Pond CD, Magin PJ, Goode SM, McElduff P, Stocks NP. Diagnosis and disclosure of a memory problem is associated with quality of life in community based older Australians with dementia. Int Psychogeriatr. 2012;24(12):1962–71.
14. Greenway-Crombie A, Snow P, Disler P, Davis S, Pond D. Influence of rurality on diagnosing dementia in Australian general practice. Aust J Prim Health. 2012;18(3):178–84.
15. Bailie C, Matthews V, Bailie J, Burgess P, Copley K, Kennedy C, et al. Determinants and Gaps in Preventive Care Delivery for Indigenous Australians: A Cross-sectional Analysis. Front Public Health. 2016;4 Available from: https://www.frontiersin.org/articles/10.3389/fpubh.2016.00034/full. [cited 2019 Jun 18].
16. Health AGD of. National Aboriginal and Torres Strait Islander Health Plan 2013-2023 [Internet]. Australian Government Department of Health; [cited 2018 Dec 3]. Available from: http://www.health.gov.au/natsihp.
17. Garvey G, Simmonds D, Clements V, O'Rourke P, Sullivan K, Gorman D, et al. Making sense of dementia: understanding amongst indigenous Australians. Int J Geriatr Psychiatry. 2011;26(6):649–56.
18. Mukadam N, Cooper C, Kherani N, Livingston G. A systematic review of interventions to detect dementia or cognitive impairment. Int J Geriatr Psychiatry. 2015;30(1):32–45.
19. Pond CD, Brodaty H, Stocks NP, Gunn J, Marley J, Disler P, et al. Ageing in general practice (AGP) trial: a cluster randomised trial to examine the effectiveness of peer education on GP diagnostic assessment and management of dementia. BMC Fam Pract. 2012;13(1):12.
20. Furler JS, Blackberry ID, Walker C, Manski-Nankervis J-A, Anderson J, O'Neal D, et al. Stepping up: a nurse-led model of care for insulin initiation for people with type 2 diabetes. Fam Pract. 2014;31(3):349–56.
21. McDermott RA, Schmidt B, Preece C, Owens V, Taylor S, Li M, et al. Community health workers improve diabetes care in remote Australian indigenous communities: results of a pragmatic cluster randomized controlled trial. BMC Health Serv Res. 2015;15:68.
22. Marley JV, Atkinson D, Kitaura T, Nelson C, Gray D, Metcalf S, et al. The be our ally beat smoking (BOABS) study, a randomised controlled trial of an intensive smoking cessation intervention in a remote aboriginal Australian health care setting. BMC Public Health. 2014;14(1):32.
23. Stoneman A, Atkinson D, Davey M, Marley JV. Quality improvement in practice: improving diabetes care and patient outcomes in aboriginal community controlled health services. BMC Health Serv Res. 2014;14(1):481.
24. Gardner KL, Dowden M, Togni S, Bailie R. Understanding uptake of continuous quality improvement in indigenous primary health care: lessons from a multi-site case study of the audit and best practice for chronic disease project. Implement Sci IS. 2010;5:21.
25. Woods C, Carlisle K, Larkins S, Thompson SC, Tsey K, Matthews V, et al. Exploring Systems That Support Good Clinical Care in Indigenous Primary Health-care Services: A Retrospective Analysis of Longitudinal Systems Assessment Tool Data from High-Improving Services. Front Public Health. 2017;5 Available from: https://www.frontiersin.org/articles/10.3389/fpubh.2017.00045/full. [cited 2019 Jun 18].
26. Spiegelman D. Evaluating public health interventions: 2. Stepping up to routine public health evaluation with the stepped wedge design. Am J Public Health. 2016;106(3):453–7.
27. Hemming K, Haines TP, Chilton PJ, Girling AJ, Lilford RJ. The stepped wedge cluster randomised trial: rationale, design, analysis, and reporting. BMJ. 2015;350:h391.
28. Larkins S, Carlisle K, Turner N, Taylor J, Copley K, Cooney S, et al. 'At the grass roots level it's about sitting down and talking': exploring quality improvement through case studies with high-improving aboriginal and Torres Strait islander primary healthcare services. BMJ Open. 2019;9(5):27–568.
29. GOLDMANN DA. Counterpoint: Sustaining CQI. International journal for Quality in health care. 1997;9(1):7–9.
30. Laycock A, Harvey G, Percival N, Cunningham F, Bailie J, Matthews V, et al. Application of the i-PARIHS framework for enhancing understanding of interactive dissemination to achieve wide-scale improvement in indigenous primary healthcare. Health Res Policy Syst. 2018;16(1):117.

31. Kitson A, Harvey G. PARIHS re-visited: introducing the i-PARIHS framework. Implementing Evidence-Based Practice in Healthcare. In: Implementing Evidence-Based Practice in Healthcare. Abingdon: Routledge; 2015. p. 41–62.
32. ISA Healthcare - MMEx. Available from: http://www.isahealthcare.com/Products/MMEx.aspx. [cited 2019 Jun 18].
33. Communicare. Telstra Corp. 2018;.
34. Australia M. Medical Benefits Scheme Health assessment for Aboriginal and Torres Strait Islander people (MBS Item 715) http://www.health.gov.au/internet/main/publishing.nsf/Content/mha_resource_kit. Accessed; 2014.
35. RACGP - QI&CPD. Available from: https://www.racgp.org.au/education/professional-development/qi-cpd. [cited 2019 Jun 19].
36. Royal Australian College of General Practitioners. Standards for general practices. South Melbourne: Royal Australian College of General Practitioners; 2010.
37. Support for Aboriginal and Torres Strait Islander people. [cited 2019 Jun 24]. Available from: https://www.myagedcare.gov.au/support-aboriginal-and-torres-strait-islander-people.
38. Hsu C-C, Sanford B. The Delphi Technique: Making Sense of Consensus. Pract Assesssment Res Evluation. 2007;12(10):1–8.
39. LoGiudice D, Smith K, Thomas J, Lautenschlager NT, Almeida OP, Atkinson D, Flicker L. Kimberley Indigenous Cognitive Assessment tool (KICA): development of a cognitive assessment tool for older Indigenous Australians. International Psychogeriatrics. 2006;18(2):269–80.
40. Sunderland T, Hill J, Mellow A, Lawlor B, Gundersheimer J, Newhouse P, et al. Clock drawing in alzheimer's disease: a novel measure of dementia severity. JAGS. 1989;37:725–9.
41. D'Elia LF, Satz P, Uchiyama CL, White T. Color trails test. Professional manual. Odessa: Psychological Assessment Resources; 1996.
42. Smith A. Symbol digit modalities test. Western Psychological: California USA; 1973.
43. Steckler A, McLeroy KR, Goodman RM, Bird ST, McCormick L. Toward integrating qualitative and quantitative methods: an introduction. Health Educ Q. 1992 Apr;19(1):1–8.
44. Angell B, Muhunthan J, Eades A-M, Cunningham J, Garvey G, Cass A, et al. The health-related quality of life of indigenous populations: a global systematic review. Qual Life Res Int J Qual Life Asp Treat Care Rehabil. 2016;25(9):2161–78.
45. Smith K, Flicker L, Lautenschlager NT, Almeida OP, Atkinson D, Dwyer A, et al. High prevalence of dementia and cognitive impairment in indigenous Australians. Neurology. 2008;71(19):1470–3.
46. Turner NN, Taylor J, Larkins S, Carlisle K, Thompson S, Carter M, et al. Conceptualizing the Association Between Community Participation and CQI in Aboriginal and Torres Strait Islander PHC Services: Qual Health Res. 2019 ; Available from: https://journals.sagepub.com/doi/abs/10.1177/1049732319843107. [cited 2019 May 2].
47. LoGiudice DC, Smith K, Shadforth G, Lindeman M, Carroll E, Atkinson D, et al. Lungurra Ngoora--a pilot model of care for aged and disabled in a remote aboriginal community--can it work? Rural Remote Health. 2012;12:2078.
48. Gardner K, Sibthorpe B, Chan M, Sargent G, Dowden M, McAullay D. Implementation of continuous quality improvement in aboriginal and Torres Strait islander primary health care in Australia: a scoping systematic review. BMC Health Serv Res. 2018;18(1):541.
49. Bailie J, Laycock A, Matthews V, Peiris D, Bailie R. Emerging evidence of the value of health assessments for aboriginal and Torres Strait islander people in the primary healthcare setting. Aust J Prim Health. 2019;25(1):1.
50. Charlson ME, Pompei P, Ales KL, MacKenzie CR. A new method of classifying prognostic comorbidity in longitudinal studies: development and validation. J Chronic Dis. 1987;40(5):373–83.
51. Reinert DF, Allen JP. The alcohol use disorders identification test: an update of research findings. Alcohol Clin Exp Res. 2007;31(2):185–99.
52. Herdman M, Gudex C, Lloyd A, Janssen MF, Kind P, Parkin D, et al. Development and preliminary testing of the new five-level version of EQ-5D (EQ-5D-5L). Qual Life Res. 2011;20(10):1727–36.
53. Smith K, Gilchrist L, Bessarab D, LoGiudice D, Flicker L, Ratcliffe J, et al. Good spirit, good life: a quality of life tool for aboriginal Australians with cognitive impairment and dementia. Alzheimers Dement J Alzheimers Assoc. 2018;14(7):1490–1.
54. Topolski TD, LoGerfo J, Patrick DL, Williams B, Walwick J, MMB P. The Rapid Assessment of Physical Activity (RAPA) Among Older Adults. Prev Chronic Dis. 2006;3(4) Available from: https://www.ncbi.nlm.nih.gov/pmc/articles/PMC1779282/. [cited 2019 Jun 24].
55. Cwikel JG, Fried AV, Biderman A, Galinsky D. Validation of a fall-risk screening test, the elderly fall screening test (EFST), for community-dwelling elderly. Disabil Rehabil. 1998;20(5):161–7.
56. Avery K, Donovan J, Peters TJ, Shaw C, Gotoh M, Abrams P. ICIQ: a brief and robust measure for evaluating the symptoms and impact of urinary incontinence. Neurourol Urodyn. 2004;23(4):322–30.
57. Roth T, Zammit G, Kushida C, Doghramji K, Mathias SD, Wong JM, et al. A new questionnaire to detect sleep disorders. Sleep Med. 2002;3(2):99–108.
58. Almeida OP, Flicker L, Fenner S, Smith K, Hyde Z, Atkinson D, et al. The Kimberley assessment of depression of older indigenous Australians: prevalence of depressive disorders, risk factors and validation of the KICA-dep scale. PLoS One. 2014;9(4):e94983.
59. Radloff LS. The CES-D scale: a self-report depression scale for research in the general population. Appl Psychol Meas. 1977;1(3):385–401.
60. Byrne G, Pachana N. Development and validation of a short form of the geriatric anxiety inventory--the GAI-SF. Int Psychogeriatr. 2011;23(1):125–31.
61. Kowal E, Gunthorpe W, Bailie RS. Measuring emotional and social wellbeing in aboriginal and Torres Strait islander populations: an analysis of a negative life events scale. Int J Equity Health. 2007;6(1):18.
62. Rubenstein L, Harker J, Salva A, Gulgoz Y, Vellas B. Screening for Undernutrition in Geratric practice: developing the short-form mini nutritional assessment (MNA- SF). J Gerontol. 2001;56(6):366–77.
63. Fried LP, Tangen CM, Walston J, Newman AB, Hirsch C, Gottdiener J, et al. Frailty in older adults evidence for a phenotype. J Gerontol Ser A. 2001;56(3):146–57.
64. Smith K, Flicker L, Atkinson D, Dwyer A, Lautenschlager NT, Thomas J, et al. The KICA Carer: informant information to enhance the Kimberley indigenous cognitive assessment. Int Psychogeriatr. 2016;28(1):101–7.
65. Higginson IJ, Gao W, Jackson D, Murray J, Harding R. Short-form Zarit caregiver burden interviews were valid in advanced conditions. J Clin Epidemiol. 2010;63(5):535–42.
66. Zarit SH, Bach-Peterson J, Reever KE. Burden interview. The gerontologist; 1980.

Publisher's Note

Springer Nature remains neutral with regard to jurisdictional claims in published maps and institutional affiliations.

Ready to submit your research? Choose BMC and benefit from:

- fast, convenient online submission
- thorough peer review by experienced researchers in your field
- rapid publication on acceptance
- support for research data, including large and complex data types
- gold Open Access which fosters wider collaboration and increased citations
- maximum visibility for your research: over 100M website views per year

At BMC, research is always in progress.

Learn more biomedcentral.com/submissions

BMC
